# *In vitro* evolution of *Pseudomonas aeruginosa* AA2 biofilms in the presence of cystic fibrosis lung microbiome members

**DOI:** 10.1038/s41598-019-49371-y

**Published:** 2019-09-06

**Authors:** Eva Vandeplassche, Andrea Sass, Astrid Lemarcq, Ajai A. Dandekar, Tom Coenye, Aurélie Crabbé

**Affiliations:** 1 Laboratory of Pharmaceutical Microbiology, Ghent University, Ghent, Belgium; 2 Department of Medicine/Department of Microbiology, University of Washington, Washington, USA

**Keywords:** Experimental evolution, Biofilms, Microbiome

## Abstract

In cystic fibrosis (CF) airways, the opportunistic pathogen *Pseudomonas aeruginosa* evolves from an acute to a chronic infection phenotype. Yet, the *in vivo* factors influencing the evolutionary trajectory of *P*. *aeruginosa* are poorly understood. This study aimed at understanding the role of the CF lung microbiome in *P*. *aeruginosa* evolution. Therefore, we investigated the *in vitro* biofilm evolution of an early CF *P*. *aeruginosa* isolate, AA2, in the presence or absence of a synthetic CF lung microbiome. Whole genome sequencing of evolved populations revealed mutations in quorum sensing (QS) genes (*lasR*, *pqsR*) with and without the microbiome. Phenotypic assays confirmed decreased production of the QS molecule 3-O-C_12_-homoserine lactone, and QS-regulated virulence factors pyocyanin and protease. Furthermore, a mixture of *lasR* and *lasR pqsR* mutants was found, in which double mutants showed less pyocyanin and protease production than *lasR* mutants. While the microbial community did not influence the production of the tested *P*. *aeruginosa* virulence factors, we observed a trend towards more mutations in the transcriptional regulators *gntR* and *mexL* when *P*. *aeruginosa* was grown alone. *P*. *aeruginosa* developed resistance to β-lactam antibiotics during evolution, when grown with and without the microbiome. In conclusion, in an experimental biofilm environment, the early *P*. *aeruginosa* CF isolate AA2 evolves towards a CF-like genotype and phenotype, and most studied evolutionary adaptations are not impacted by CF microbiome members.

## Introduction

*Pseudomonas aeruginosa* is a Gram-negative, versatile, opportunistic pathogen that causes chronic lung infections in cystic fibrosis (CF) patients^1–3^. Because of its large genome – over 6 Mb and almost 6000 genes – it is able to quickly adapt to, and hence thrive in, different environments and stress conditions^4,5^. In the CF airways, a characteristic evolutionary adaption of *P*. *aeruginosa* is observed with a gradual transition from an ‘acute’ phenotype (characterized by motility, protease production, and type III secretion) to a ‘chronic’ phenotype (characterized by biofilm formation, decreased antibiotic susceptibility, loss of virulence factor production, altered pro-inflammatory effect, and hypermutability)^5–12^. This adaptation is thought to be due to a combination of selective pressures present in the CF lungs, i.e. viscous mucus, hyperinflammation, prolonged antibiotic treatment, the presence of bacteriophages and a complex microbial community^4,7^. Moreover, an intra-patient phenotypic diversity of *P*. *aeruginosa* isolates has been observed in CF patients^13–16^, which is possibly due to regional adaptation in the spatially heterogeneous lung environment^5,17,18^.

One of the key features of *P*. *aeruginosa* evolution in the CF lung is the emergence of mutations in the *las* quorum sensing (QS) system, which are associated with a loss in production of virulence factors, such as proteases and exotoxins^19–21^. QS in *P*. *aeruginosa* consists of multiple interrelated signalling circuits. There are three acyl-homoserine lactone responsive transcription factors, LasR, RhlR, and QscR; and a quinolone-responsive transcripton factor called PqsR. The *las* system sits at the top of this complex hierarchy^22^. Multiple other pathoadaptive mutations have also been associated with chronic *P*. *aeruginosa* infection, such as mutations in *gacS*, *retS*, *rpoN*, *ampR*, *mexT* and *mucA*^5^. During *in vitro* planktonic and biofilm evolution studies, mutations in the *lasR* gene, coding for the transcriptional regulator of the *las* QS system, have repeatedly been observed and these have been associated with increased antibiotic resistance^8,23–26^. However, most of these studies have focused solely on the evolution of *P*. *aeruginosa* in single culture, and do not take into account the complex bacterial community present in the CF airways *in vivo*^27–29^. This CF lung microbiome can contain various pathogens besides *P*. *aeruginosa* (e.g. *Staphylococcus aureus*, *Haemophilus influenzae*. *Burkholderia cenocepacia*, *Achromobacter xylosoxidans*, *Streptococcus milleri* group bacteria and *Stenotrophomonas maltophilia*) as well as other non-pathogenic bacteria (including *Rothia mucilaginosa* and *Gemella haemolysans*)^28,30–32^. However, whether the characteristic phenotypic and genotypic adaptation of *P*. *aeruginosa* in the course of chronic infection of the respiratory tract of people with CF, including hallmark mutations in the QS system, is influenced by the presence of this complex microbial community is unclear. This is of particular importance since the QS system is involved in the competitive behaviour of *P*. *aeruginosa*^33,34^. Production of many virulence factors, such as pyocyanin, hydrogen cyanide, elastase and rhamnolipids, that exert antimicrobial activity against other bacteria, are QS-regulated^33,35–38^. In addition, QS partially contributes to the regulation of siderophores (such as pyoverdine) that are important in competition for iron.

We performed a biofilm evolution study using the early CF *P*. *aeruginosa* isolate AA2. *P*. *aeruginosa* was cultured *in vitro* in a biofilm during 54 days (18 cycles of 72 h biofilm formation) in the absence and presence of a microbial community comprised of commonly isolated species in the CF lung. Whole genome sequencing (WGS) was performed to unravel genotypic differences and phenotypic tests were carried out to investigate changes in production of various virulence factors (i.e. phenazines, proteases, rhamnolipids), antibiotic susceptibility, growth, motility, and competitive behaviour. Furthermore, the pro-inflammatory response of human epithelial cells to evolved versus unevolved *P*. *aeruginosa* strains was examined in a differentiated 3-D lung epithelial cell model.

## Materials and Methods

### Bacterial strains and culturing conditions

Bacterial strains used are shown in Table S1 and were cultured on solid media as described before^39^. For the evolution study, liquid cultures of each bacterial strain were grown until stationary phase in BHI broth (37 °C, 250 rpm; Table S1). *S*. *anginosus* and *G*. *haemolysans* cultures were incubated in microaerophilic conditions (CampyGen Compact system, Thermo Fisher Scientific, USA).

For phenotypic testing and sequencing of evolved populations originating from the biofilm evolution experiment, an aliquot of the frozen biofilm (stored as glycerol stock at −80 °C) was plated on LB agar and incubated overnight at 37 °C. The entire growth on the agar plate was then divided into two Microbank cryovials with beads (Pro-Lab Diagnostics) and frozen at −80 °C. A single bead was then used to inoculate 5 ml LB broth (incubated at 37 °C, 250 rpm, 16 hours) to serve as inoculum for phenotypic tests and DNA extraction. These cultures are designated “evolved populations” throughout the text for the sake of clarity.

### Biofilm evolution model set-up

**Figure 1:**
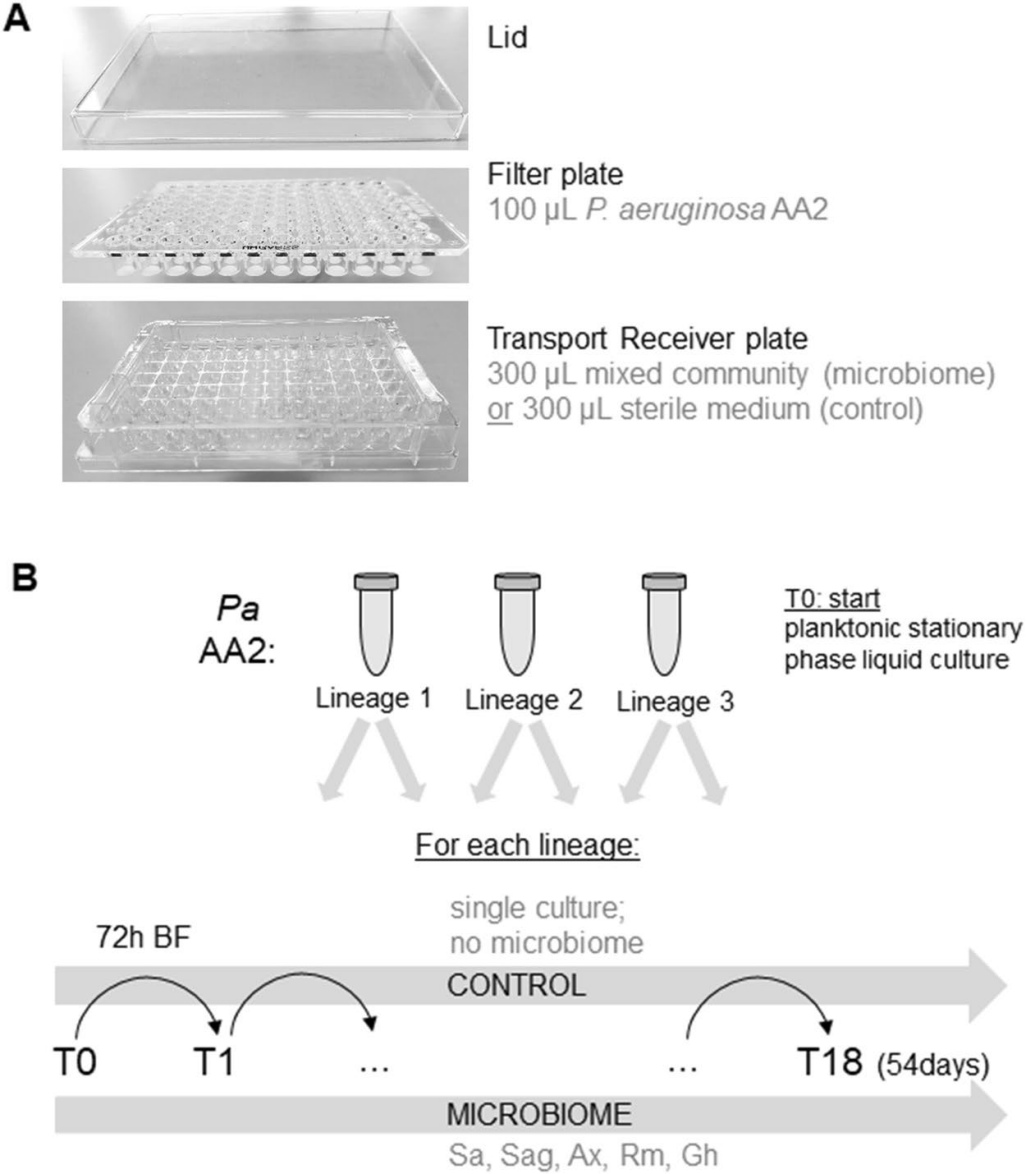
Multiscreen evolution model set-up and experiment overview. (**A)** The bottom transport receiver plate was sterilized by exposure to UV-light and contained either medium (control condition) or a mixed bacterial community of *S*. *aureus*, *S*. *anginosus*, *A*. *xylosoxidans*, *R*. *mucilaginosa*, and *G*. *haemolysans* (microbiome condition). The sterile (0.22 µm PDFV) filter plate contained *P*. *aeruginosa* AA2 and was placed in the receiver plate, with the filters being emerged in the medium. A sterile lid secluded the set-up. (**B**) Pa AA2: *P*. *aeruginosa* AA2. Three independent overnight cultures were used to start three independent evolution samples = lineages (L1, L2, L3). For each lineage, *P*. *aeruginosa* biofilms were evolved in the absence (control) or presence of the CF microbial community (microbiome). Sa: *S*. *aureus*, Sag: *S*. *anginosus*, Ax: *A*. *xylosoxidans*, Rm: *R*. *mucilaginosa*, and Gh: *G*. *haemolysans*. T0 is the planktonic overnight culture at the start of the experiment, T1 (timepoint 1) is the *P*. *aeruginosa* biofilm (BF) obtained after 72 hours of microaerobic growth. 18 cycles of 72 hours ended in T18 (timepoint 18) after 54 days.

A Multiscreen 96-well system (Millipore, Merck KGaA, Germany) consisting of a sterile filter plate with 0.22 µm PVDF filter membranes and a transport receiver plate (sterilized by 1 hour exposure to UV light) was used for biofilm formation of *P*. *aeruginosa* AA2 in the absence or presence of the other microbiome members (Fig. 1A). All bacterial suspensions were diluted in BHI medium supplemented with 2.5% lysed horse blood (BHI + LYS) (Biotrading, The Netherlands; lysed horse blood stock according to EUCAST protocol^40^). For the initiation of the evolution study (first cycle, T1), 100 µL of *P*. *aeruginosa* suspension (approx. 5 × 10^6^ CFUs of starting/unevolved culture) was transferred to the upper filter plate, and 300 µL of microbiome community culture (containing approx. 5 × 10^6^ CFUs of each strain, i.e. *S*. *aureus*, *S*. *anginosus*, *A*. *xylosoxidans*, *R*. *mucilaginosa*, and *G*. *haemolysans*) or 300 µL of medium (control) was transferred to the receiver plate. This way, no physical contact between *P*. *aeruginosa* and the other bacterial species occurred, yet medium containing soluble secreted products could migrate through the 0.22 µm low protein-binding filter. Then, cultures were incubated in microaerophilic conditions (±5% O_2_, ±15% CO_2_; CampyGen Compact system, Thermo Fisher Scientific) for 72 hours (3 days). After 3 days of culture, *P*. *aeruginosa* AA2 biofilm cells were removed from the filter plate. First, a wash step with physiological saline (PS; 0.9% [w/v] NaCl in MilliQ water) was performed after which two rounds of vortexing and sonication resulted in homogenized *P*. *aeruginosa* biofilm cells, as described before^39^. The colony forming units (CFU) in the biofilm were quantified by microdilution plating on LB agar. A fraction of the biofilm was stored at −80 °C (in PS containing a final concentration of 20% glycerol) until further genomic and phenotypic experiments were conducted. To initiate the second cycle and for each cycle hereafter (T2 – T18), the homogenized biofilm was diluted 1/40 in BHI + LYS (resulting in ±5 × 10^6^ CFUs) and transferred to the filter plate. The microbial community was added to the transport receiver plate as described above for the first cycle. This was repeated 18 cycli representing a total of 54 days. Three independent lineages were cultured in parallel which were started from three different *P*. *aeruginosa* AA2 stationary phase cultures; each lineage was cultured in the presence and absence of the microbiome members (Fig. 1B).

### DNA extraction and sequencing

#### Genomic DNA extraction: glass-bead protocol

Genomic DNA was extracted from 2 mL planktonic stationary phase cultures of the unevolved strain (T0, initial culture) or evolved populations of *P*. *aeruginosa* AA2. After centrifugation, the pellet was resuspended in 200 µL TE buffer (10 mM Tris-HCl pH 8 + 1 mM EDTA pH 8). Of this, 100 µL was added to lysis tubes containing 500 µL acid-washed glass beads (Sigma-Aldrich, USA) and 500 µL lysis buffer (50 mM Tris-HCl pH 8, 70 mM EDTA pH 8, 1% sodium dodecyl sulfate) with 0.5 µg/mL pronase (Roche, Germany). After vortexing, the tubes were incubated at 37 °C for 1 hour and then pulse-spinned. Afterwards, 200 µL saturated ammonium acetate was added, vortexed again, and spinned for 2 minutes. 600 µL chloroform was added, the mixture was vortexed, and then centrifuged for 5 minutes to separate the phases. Then, 400 µL of the top aqueous phase was transferred to a new tube containing 1 mL 100% ethanol. This was mixed by inversion and centrifuged for 5 minutes. The supernatant was removed, washed with 70% ethanol, and air-dried. The resulting DNA was dissolved in low-EDTA-TE buffer (10 mM Tris-HCl pH 8 + 0.1 mM EDTA pH 8 + 0.5 µg/mL RNase A (Qiagen, Germany)) and the concentration measured using the BioDrop µLITE (Isogen Life Science, The Netherlands).

#### Whole genome sequencing (WGS) and bioinformatics analysis

WGS libraries were prepared with the NEBNext kit (New England Biolabs, Ipswich, USA) and sequenced on the Illumina Nextseq 500 platform (Oxford Genomics Center, University of Oxford, United Kingdom), yielding 5 to 9 million 150 bp paired end reads per sample. Demultiplexed raw reads were imported into CLC Genomics Workbench 11.0.1 (QIAGEN Bioinformatics, Aarhus, Denmark) for all further analysis steps. Reads were quality-trimmed (error probability limit 0.05, reads < 15 nt were discarded) and the input strain sample (T0) was *de novo* assembled (word size 22, bubble size 55, mismatch cost 2, insertion and deletion cost 2, length fraction 0.5, similarity fraction 0.8), generating 92 contigs (total length 6244190, N50 = 210536, max length 471773, average coverage 123.5). The RAST annotation engine^41^ was used to annotate the consensus sequence of contigs. The reads derived from the evolved populations were then mapped to all contigs, with cut-offs 0.5 for length fraction, and 0.8 for similarity fraction for mapped reads. Mapping parameters were: match score 1, mismatch cost 2, insertion and deletion cost 3. The Basic Variant Detection tool was used to detect single nucleotide polymorphisms (SNPs) with a minimum coverage of 10 and a minimum reference-to-variant ratio of 35%. Genes that were mutated in at least one sample at a frequency above 35% in T18 were then further analysed in T0, T1, T10 and T18 using the Low Frequency Variant Analysis tool of CLC Genomics Workbench at a frequency threshold of 2%. All SNPs were then manually screened for false positives in regions containing repetitive sequences or hairpins, which caused poor mapping, or for erroneous reads. The InDels and Structural Variants tool was used to detect insertions and deletions, with a p-value threshold of 0.0001. The output was manually screened on mapping patterns of un-aligned read ends and only entries with a single breakpoint and identical sequences in the un-aligned read ends were reported. The consensus sequence of the un-aligned read ends was then used to confirm the deletion or to identify the nature of the inserted sequence. The sequencing raw data is deposited at Array Express under accession number E-MTAB-7331.

#### Sanger sequencing

To verify mutations observed in the WGS data, Sanger sequencing of the *lasR* and *pqsR* genes of 10 isolates obtained from single colonies was performed. Two primer pairs (Table S2) that amplified 900–1200 base pair regions were designed targeting the mutated region identified by WGS. After PCR amplification, the amplicons were purified using the NucleoSpin Gel and PCR Clean-up kit (Macherey-Nagel, Germany) and sequenced at GATC Biotech (Eurofins Genomics, Germany). Resulting sequences were then inspected visually for specific mutations using the Chromas software program (Technelysium Pty Ltd, Australia).

### Bacterial growth curves

To compare planktonic growth of all *P*. *aeruginosa* AA2 populations, liquid stationary phase cultures were diluted to approx. 5 × 10^5^ CFU/mL (based on OD_590nm_) in LB. Of each culture, 100 µL was transferred to a PVC 96-well plate (Thermo Fisher Scientific) in triplicate. The 96-well plate was incubated statically at 37 °C in the EnVision Multilabel Plate Reader (Perkin Elmer, USA) and OD_590nm_ was measured every 30 min for 24 hours. After 24 hours, end-point cultures were diluted and plated on LB agar to determine the number of CFU.

### Quantification of biofilm colony forming units and biomass

*P*. *aeruginosa* cultures were diluted in LB medium to OD_590nm_ 0.08 (corresponding to ±5 × 10^7^ CFU/mL), where after 100 µL of each culture was transferred in triplicate to the wells of a flat PVC 96-well plate, and then incubated at 37 °C for 24 hours. Afterwards, biofilms were washed and harvested in PS by two cycles of vortexing (5 min, 900 rpm) and sonication (5 min; Branson Ultrasonic bath, Hach Company, USA). The resulting cell suspensions were diluted and plated on LB plates; after overnight incubation at 37 °C the number of CFU was determined. Biomass of biofilms was quantified by crystal violet staining as described previously^42,43^.

### Antibiotic susceptibility testing

Minimal inhibitory concentrations (MIC) for ceftazidime (Sigma-Aldrich), aztreonam (TCI Europe), ciprofloxacin (Sigma-Aldrich), colistin (TCI Europe) and tobramycin (TCI Europe) were determined according to the EUCAST guidelines by the microdilution broth method as described elsewhere^44,45^.

### Biofilm competition experiment

*P*. *aeruginosa* cultures were co-cultured with *S*. *aureus* for 24 hours. Both were diluted in LB medium to OD_590nm_ 0.08 and 0.2 respectively, corresponding to ±5 × 10^7^ CFU/mL. 100 µL of mixed culture (50 µL of each) was transferred in triplicate to the wells of a flat PVC 96-well plate, and then incubated at 37 °C for 24 hours. Afterwards, the resulting biofilms were loosened and diluted as described above, and plated onto selective media to quantify the separate strains. *P*. *aeruginosa* and *S*. *aureus* were quantified on LB medium supplemented with 1.25 mg/L triclosan and LB medium supplemented with 7.5% NaCl respectively^39^.

### Motility assays

Bacterial cultures were diluted to OD_590nm_ = 1 after which three types of motility were tested by using media with different agar concentrations: swimming (0.3%), twitching (1.5%) and swarming (0.5%) as described before^46^. Briefly, for determination of swimming and swarming motility, 1 µL of bacterial culture was spotted on the surface of LB plates with 0.3% agar or 0.8% nutrient plates with 0.5% glucose and 0.5% agar respectively. For twitching motility, regular LB agar (1.5%) plates were stab-inoculated to the bottom of the petri dish with 1 µL of bacterial suspension. All plates were incubated at 37 °C for 48 hours after which bacterial migration diameters were measured for swimming and swarming. Twitching was visualized by crystal violet staining as previously described^46^.

### Pyoverdine quantification

For pyoverdine quantification, overnight cultures were grown in LB, centrifuged, and the resulting supernatant filter-sterilized (0.22 µm). 100 µL of filtered supernatant was transferred to a flat PVC 96-well plate and absorbance at 405 nm was measured in the EnVision Multilabel Plate Reader, as previously described^47^.

### Pyocyanin quantification

For pyocyanin quantification, liquid cultures were grown in LB broth for 24 hours in a shaking warm water bad (37 °C). Supernatants were collected after centrifugation and then filter-sterilized (0.22 µm Merck-Millipore filters). Afterwards, a chloroform-HCl extraction was performed to determine pyocyanin levels, as described previously^48^.

### Rhamnolipid detection

Semi-quantitative detection of rhamnolipid production by all populations was assessed by inoculating 70 µL of bacterial suspension into wells cut out from minimal medium agar plates supplemented with 0.05 mg/L methylene blue and 2 mg/L cetyl trimethylammonium bromide (CTAB)^49,50^. Sizes of blue halos were measured after 48 hour incubation at 30 °C and incubation at 4 °C for 48 hours.

### Quantification of protease activity

Overnight cultures were grown for 16 hours in LB. Cultures were centrifuged and the supernatant filter-sterilized, where after proteolytic activity was determined using an azocasein assay^51,52^.

### Quantification of *N*-3-oxododecanoyl-L-homoserine lactone production

Homoserine lactone (HSL) signal molecules were first extracted using ethyl acetate as described previously^53^. Afterwards, 10 µL of concentrated HSL molecules was added to 100 µL overnight culture of *Escherichia coli* (stationary phase culture in LB; OD 0.3) containing the reporter plasmid pUCP22NotI-P*lasB*::*gfp*(ASV)P*lac*::*lasR* which expresses GFP (green fluorescent protein) in response to *N*-3-oxododecanoyl-L-homoserine lactone (3-O-C_12_-HSL)^54,55^. Cultures were incubated in a black 96-well plate for 24 hours at 37 °C and fluorescence was measured in the EnVision Multilabel Plate Reader. The increase in fluorescence over 24 hours is related to the amount of 3-O-C_12_-HSL present.

### Quantification of epithelial cell viability

Cytotoxicity of the bacterial populations was assessed by infection of an *in vivo*-like 3-D alveolar epithelial cell culture model (A549 cell line)^56–59^ and quantified by staining with annexin V and propidium iodide, which allows to evaluate host cell apoptosis and necrosis (Alexa Fluor® 488 Annexin V/Dead Cell Apoptosis Kit, Thermo Fisher Scientific). On the day of infection, 3-D cell aggregates were transferred to a 48-well plate at a concentration of 2.5 × 10^5^ cells/well in GTSF-2 medium (Hyclone, USA) supplemented with 1.5 g/L sodium bicarbonate (Sigma-Aldrich) and 2.5 mg/L insulin transferring sodium selenite (Lonza). The bacterial suspension was then added to each well, to obtain a targeted MOI (multiplicity of infection) of 10:1. After 6 hours of infection, epithelial cells were washed with HBSS (Hank’s Balanced Salt Solution, Life Technologies, Thermo Fisher Scientific) and dissociated from the microcarrier bead scaffolds by incubation with 250 µL trypsin (0.25%, Life Technologies) for 3 minutes followed by thorough pipetting. The trypsinisation reaction was stopped by adding 250 µL GTSF-2 medium with 10% fetal bovine serum (FBS) (Life Technologies, Thermo Fisher Scientific). Cells were then harvested by centrifugation and subsequently stained by adding 5 µL Alexa Fluor® 488 annexin V and 2 µL propidium iodide (100 µg/mL), according to the manufacturer’s instructions. Viability of cells – differentiating between live, necrotic, and apoptotic cells – was analysed by flow cytometry (Attune NxT Flow Cytometer, Thermo Fisher Scientific), using excitation at 488 nm and measuring fluorescence emission at 530 nm and 575 nm for annexin V and PI, respectively.

### Quantification of the NF-κB - mediated inflammatory response

The NF-κB – mediated inflammatory response was tested in the above described *in vivo*-like 3-D alveolar epithelial cell culture model, using NF-κB-luciferase-transfected A549 cells (BPS Bioscience, USA). Bacterial cultures and 3-D aggregates were diluted in the above-described cell culture medium to result in an MOI of 10:1 and transferred to a 48-well plate. After 4 hours of incubation, the supernatant was removed and the 3-D aggregates were transferred to a black PVC 96-well plate. The epithelial cells were then lysed and the luciferase substrate added using the ONE-Step Luciferase Assay system (BPS Bioscience) according to the manufacturer’s instructions. Luminescence was measured in the EnVision Multilabel Plate Reader.

### Statistical analysis

Statistical analysis was performed in SPSS 24.0. Normality of the data was examined via the Shapiro-Wilk test. Normally distributed data were then assessed by an independent samples t-test or one-way ANOVA. Not normally distributed data were evaluated using a Mann-Whitney or Kruskal-Wallis non-parametric test. Statistical significance of data is assumed when p-values are ≤0.05.

## Results

### *P*. *aeruginosa* AA2 biofilm evolution model

Biofilms grown in the filter plate model (Fig. 1) were investigated at different time points (24 h, 48 h, 72 h) to determine how long it was possible to co-culture all microbiome members (i.e. without losing one or more). As all members of the CF synthetic microbiome remained present at the tested time points (Fig. S1), the 72 h co-culture time was selected.

At the end of each 72 h cycle (18 in total), *P*. *aeruginosa* AA2 biofilm cells were harvested directly from the filter plate and CFUs were immediately determined. For two out of three lineages (L1 and L3), a trend towards increased biofilm formation over time was observed, regardless of whether other species were present (Fig. S2). When pooling the data of the three lineages, a significant increase in biofilm formation was observed when comparing the first (T1) and last (T18) cycle, both in the presence and absence of the microbiome (p < 0.01) (Fig. S8A).

We also determined biofilm formation in a conventional PVC microtiter plate starting from preserved cultures of the unevolved strain (T0) or evolved populations (T1, T18) of *P*. *aeruginosa* AA2, using plating and crystal violet staining of biomass (Fig. S3). The evolved populations (T18) did not show significant increases in biofilm formation after 24 hours in LB. However, when pooling the data of the three lineages, a significant decrease in biofilm formation was observed between T1 and T18 (p < 0.01) both with and without the microbiome (Fig. S8B). Biomass was also significantly reduced in T18 compared to T1 for the pooled samples without the microbiome (p < 0.01) (Fig. S8C).

### *LasR* and *pqsR* mutations occur in the presence and absence of the CF microbial community

At the beginning (T1), middle (T10) and at end of the evolution experiment (T18), WGS was performed for all evolved *P*. *aeruginosa* populations and these sequences were compared to that of the unevolved T0 strain (Table S3). Only six genes were found to be mutated at a frequency of ≥ 35% at T18 (Table 1) in at least one lineage, all of these were present in at least two lineages when the lower threshold for frequency was applied. Nine genes were mutated at < 35% frequency at T18 (Table S4), and none of these occurred in more than one lineage.

**Table 1:** Whole genome sequencing data for evolved (T18) *P*. *aeruginosa* populations compared to the starting planktonic culture strain (T0).

Sample name	Gene name	PA number	Mutation type	Nucleotides	Position (contig) nt	Amino acids affected	Relative abundance (%)
T18 L1 C	*lasR*	PA1430	Del	ΔG	(2) 294159	K182RfsX27^*a^	100
	*pqsR*	PA1003	In In	+A transposase	(11) 20879 (11) 20654	L98FfsX34 H173transpos^*b^	81 5
	*gntR*	PA4132	SNP SNP SNP	C → G A → C G → A	(34) 41825 (34) 41176 (34) 40992	Y349X D133A V72M	41 4 2
	*mexL*	PA3678	SNP	G → A	(12) 221690	A26T	23
	*sahH*	PA0432	SNP	G → A	(1) 95660	297 nt upstream^*c^	99
	*pilA*	PA4525	Del	CTACCA	(6) 118729	T154_T155del	97
T18 L1 M	*lasR*	PA1430	Del	ΔG	(2) 294159	K182RfsX27	100
	*pqsR*	PA1003	Del In Del	ΔTTGAT + A ΔCGAGCTGACCGC	(11) 20762 (11) 20879 (11) 21061	I136AfsX208 L98FfsX34 A34_S37del^*d^	69 11 7
	*sahH*	PA0432	SNP	G → A	(1) 95660	297 nt upstream	56
	*pilA*	PA4525	Del	CTACCA	(6) 118729	T154_T155del	92
T18 L2 C	*lasR*	PA1430	In	+A	(2) 294001	S129KfsX104	94
	*pqsR*	PA1003	SNP	C → T	(11) 20505	D223H	38
	*mexL*	PA3678	duplication SNP SNP	35 nt T → G C → A	(12) 222153 (12) 221786 (12) 222105	E192GfsX0 F58T A164D	24 17 12
	*sahH*	PA0432	Del	ΔG	(1) 95633	271 nt upstream	32
	*pilA*	PA4525	Del	CTACCA	(6) 118729	T154_T155del	98
T18 L2 M	*lasR*	PA1430	In Del	+A ΔG	(2) 294001 (2) 294159	K182RfsX27	59 31
	*pqsR*	PA1003	SNP	A → T	(11) 21047	L42Q	59
	*pqsA-phnA*	PA0996-PA1001	Del	Δ(4618 nt)	(11) 13454–18072	V306(pqsA)_S2(phnA) del fsX69^*e^	10
	*mexL*	PA3678	SNP	G → C	(12) 221695	K27N	23
	*pilA*	PA4525	Del	CTACCA	(6) 118729	T154_T155del	49
T18 L3 C	*lasR*	PA1430	In Del	+A ΔG	(2) 294001 (2) 294159	S129KfsX104 K182RfsX27	89 10
	*pqsR*	PA1003	In SNP	+G T → G	(11) 20642 (11) 20645	L178VfsX168 H176P	74 9
	*gntR*	PA4132	SNP In In	A → C + CTG + C	(34) 41988 (34) 41357 (34) 40926	T404P L194_L195insL^*f^ G52RfsX260	9 9 2
	*mexL*	PA3678	SNP SNP SNP	T → G G → A G → T	(12) 222042 (12) 221744 (12) 221842	M143R A44T Q76H	46 9 6
	*sahH*	PA0432	SNP	G → A	(1) 95660	297 nt upstream	77
	*pilA*	PA4525	Del	CTACCA	(6) 118729	T154_T155del	87
T18 L3 M	*lasR*	PA1430	Del In	ΔG + A	(2) 294159 (2) 294001	K182RfsX27 S129KfsX104	89 12
	*pqsR*	PA1003	In Del	transposase ΔAG	(11) 21137 (11) 20400	F12transpos Y258TfsX131	33 5
	*sahH*	PA0432	SNP	G → A	(1) 95660	297 nt upstream	10
	*pilA*	PA4525	Del	CTACCA	(6) 118729	T154_T155del	93

In: insertion (+), Del: deletion (Δ), SNP: single nucleotide polymorphism (→). nt: nucleotide. QS: quorum sensing. T18: timepoint 18 (final evolved population). L1, L2, L3: lineage 1, 2, 3. C: control (=evolution in the absence of the microbiome), M: microbiome (=evolution in the presence of the microbiome). PA number: *Pseudomonas aeruginosa* PAO1 homologue number. This table lists genes that were mutated in at least one sample at a frequency above 35%.

^*a^K182RfsX27: K at position 182 is the first amino acid residue affected, it is changed to R followed by a frameshift of 27 codons in total, including the next stop codon.

^*b^H173transpos: the codon H at position 173 is interrupted by a transposase.

^*c^The mutations relating to *sahH* are located in the intergenic region upstream of the start of the gene.

^*d^A34_S37del: the amino acids 34 to 37 are deleted.

^*e^V306(pqsA)_S2(phnA)del fsX69: the deletion affects V in position 306 of pqsA to S in position 2 of phnA, followed by a frameshift of 69 codons including the stop codon.

^*f^L194_L195insL: L is inserted between position 194 and 195.

In all *P*. *aeruginosa* populations that evolved in the presence and absence of the CF microbial community, mutations were found in the *lasR* QS regulator gene (*P*. *aeruginosa* PAO1 homologue designation: PA1430), with a frequency (relative abundance of affected reads in the population) of up to 100% already present at T1 (Table S3). Re-mapping the reads for T0 strain to the AA2 assembly revealed that mutations in *lasR* were already present with a cumulative frequency of 33% in the starting culture (Table S3). These mutations in *lasR* were the only mutations present in T0 or T1.

Mutations in *pqsR* (PA1003), another QS regulator gene, were found in all samples at frequencies of up to 93% at T10 and T18, these were not found at T1 or T0 (Tables 1, S3). The types of mutations in *lasR* and *pqsR* include insertions of A/G or a transposase, several deletions, and different SNPs (single nucleotide polymorphisms).

Mutations were also found in two other transcriptional regulators. The *tetR* gene encoding MexL (PA3678, part of the TetR protein family), a specific transcriptional repressor of the multidrug efflux operon *mexJK*^60,61^, was mutated at T18 in all samples that evolved in the absence of the microbiome (at frequencies up to 61%), and only in one sample that evolved in the presence of the microbiome. Most mutations in *tetR* were SNPs. Furthermore, in two control samples grown without the microbiome (T18 L1 C; T18 L3 C), mutations in the *gntR* gene (PA4132) were found at frequencies of up to 47% at T18, which were SNPs or insertions. *gntR* presumably encodes a DNA-binding transcriptional regulator from the MocR family and contains an aminotransferase domain, although it has not been fully characterised yet. The *mexL*, *mexJK*, and *gntR* genes have all been shown to be QS-activated^22^.

A deletion was also found in *pilA* (PA4525), the major pilin of the type IV pili, in all lineages regardless of the presence of the microbiome at T10 and T18, at frequencies of up to 97% (Tables 1, S3).

Finally, in all but one (T18 L2 M) samples, a mutation (SNP of G to A, or deletion of G) upstream of *sahH* (PA0432), encoding an adenosylhomocysteinase, was found (Table 1). This mutation consistently occurred at lower frequency in the presence of the microbiome (L1: 56 vs 99%, L2: 0 vs 32%, and L3: 10 vs 77%).

Often, different types of mutations occurred in the same gene for the same sample. Yet, it could not be determined if (or what fraction of) a population contained one or multiple mutations.

Nine genes with low frequency mutations (<35%) were observed at T18 and included the surface attachment sensor SagS, two flagellar genes and a type III secretion protein (Table S4).

### 3-O-C_12_-HSL and QS-regulated virulence factor production is reduced in evolved populations

**Figure 2:**
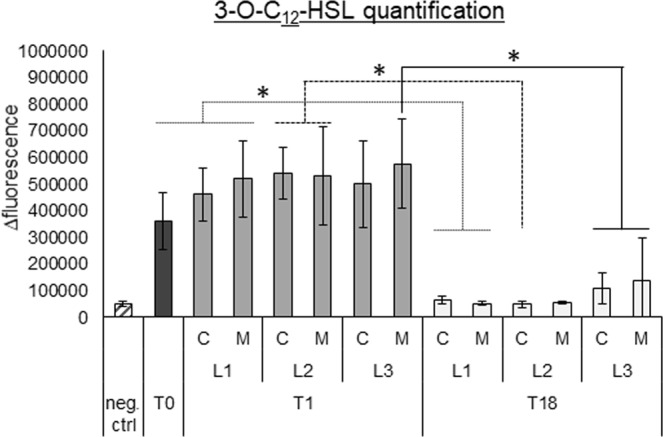
Quantification of 3-O-C_12_-HSL production using an *E*. *coli* biosensor strain (expressing GFP in response to this molecule). The data is presented as delta fluorescence, by measuring the difference in fluorescence at 24 h compared to the start of the experiment, to normalize for autofluorescence. Graphs show means, error bars indicate standard deviations. n = 3, *p ≤ 0.05. T0: timepoint 0, planktonic culture. T1: timepoint 1, 72 h biofilm cells. T18: timepoint 18, biofilm cells after 18 cycles (54 days). L1: lineage 1, L2: lineage 2, L3: lineage 3. C: control *P*. *aeruginosa* evolved without microbiome, M: *P*. *aeruginosa* evolved with microbiome. Neg. ctrl: negative control, pure *E*. *coli* culture.

After our discovery of the mutations found in the gene encoding *lasR*, we measured production of 3-O-C_12_-HSL by the T1 and evolved T18 populations using an *E*. *coli* biosensor strain expressing GFP in response to this QS molecule. Production of 3-O-C_12_-HSL was significantly reduced in the evolved populations (T18) compared to the populations at T1 in all lineages (Fig. 2). This showed that the observed mutations led to a null phenotype for the population only at T18, although *lasR* is already mutated at T1. Pooling the data from three lineages confirmed this observation (Fig. S8D).

**Figure 3:**
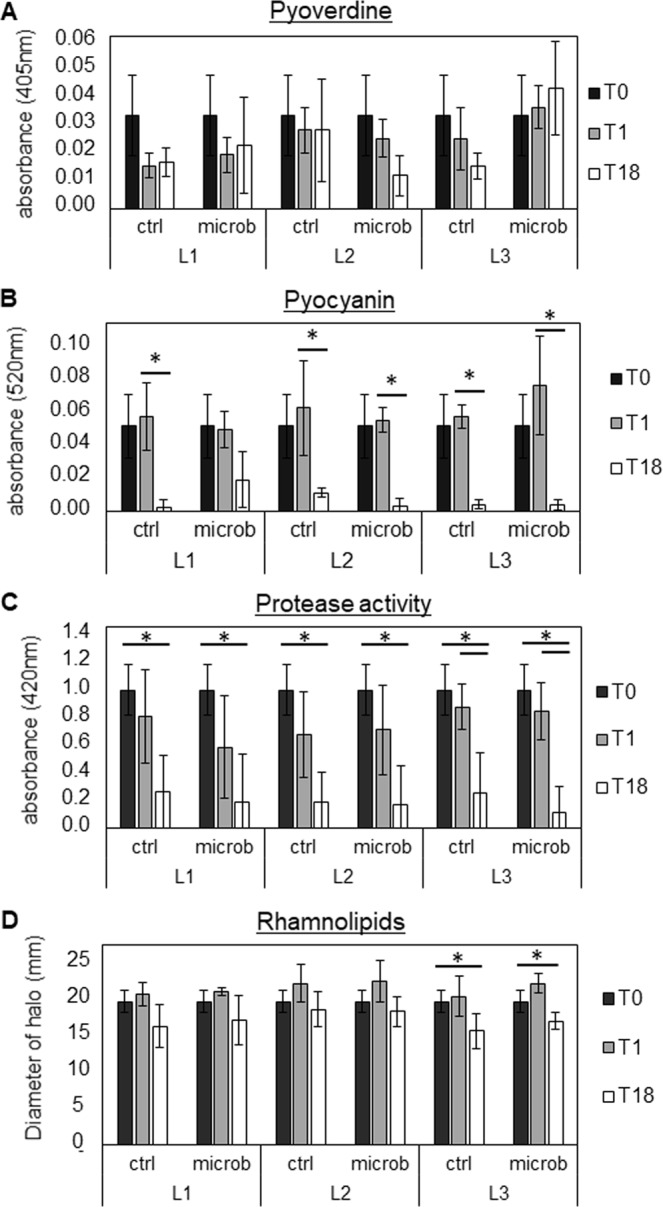
Determination of pyoverdine (**A**), pyocyanin (**B**), protease (**C**), and rhamnolipid (**D**) production by unevolved and evolved *P*. *aeruginosa* AA2  populations. Graphs show means, error bars indicate standard deviations. n ≥ 3, *p ≤ 0.05. T0: timepoint 0, planktonic culture. T1: timepoint 1, 72 h biofilm cells. T18: timepoint 18, biofilm cells after 18 cycles (54 days). L1: lineage 1, L2: lineage 2, L3: lineage 3. C: control single-culture, M: microbiome.

To evaluate whether the observed mutations in the QS system resulted in downstream differences at the phenotypic level, production of QS-regulated virulence factors was quantified (Fig. 3). Pyocyanin production and protease activity were significantly reduced in the evolved populations (T18) for all lineages compared to T0 and/or T1 (both in the absence and presence of microbiome members) (p ≤ 0.05) (Fig. 3), and when all lineages were pooled (p ≤ 0.01) (Fig. S8E,F). In the evolved population of lineage 3, rhamnolipid production was also significantly reduced (p ≤ 0.05), and when data of all lineages was pooled a significantly lower rhamnolipid production was observed between T1 and T18 (Fig. S8G). However, no significant differences in pyoverdine production were observed (Figs 3, S8H) and swimming, swarming, and twitching motility were also not affected (Figs S4 and S8I–K).

In order to examine whether the differences in virulence factor production were due to potential differences in growth, growth curves were determined for evolved and unevolved populations (Figs S5, S8L). In all lineages, no differences in growth were observed between all populations in the lag and exponential phases. However, in the stationary growth phase, significant differences in absorbance values could be observed between T0 and populations from T1 compared to the evolved populations at T18 (p ≤ 0.05). Nevertheless, this could not be linked to a change in CFUs (Figs S6, S8M).

Finally, the activation of the pro-inflammatory NF-κB pathway was evaluated following exposure of an *in vivo*-like 3-D lung epithelial model to the evolved or unevolved populations. NF-κB pathway activation was observed for all populations and no significant differences in activation were observed (p > 0.05) (Figs S7A, S8N). Host cell viability was approximately 80% for all infected samples (Figs S7B, S8O).

### A mix of *lasR* and *lasR pqsR* mutants is present in evolved biofilms

One evolved biofilm (T18 L2 C) was plated and ten single colonies (C1–C10) were investigated further for mutations in *lasR* and *pqsR* using Sanger sequencing of the region that was found to be affected by WGS. Theoretically, almost all isolates should have the mutation (insertion) in *lasR*, while only 38% should have the *pqsR* mutation (SNP), according to the WGS data. While Sanger sequencing indeed confirmed that all isolates had a mutation in the *lasR* gene (10/10, 100%), six out of ten (60%) isolates also had a *pqsR* mutation (Table S5).

### *lasR pqsR* mutants exert lower production of pyocyanin and protease than *lasR* mutants

**Figure 4:**
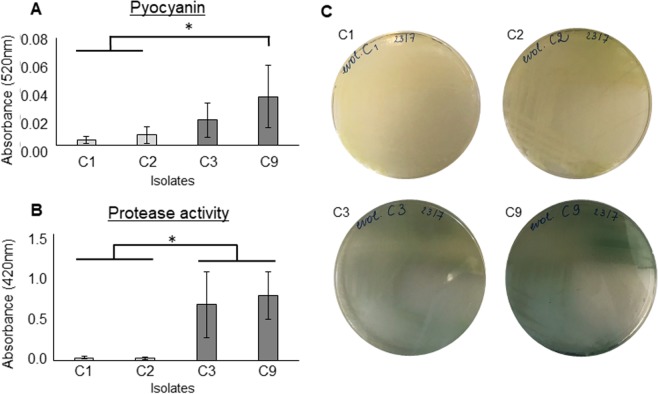
Pyocyanin production (**A**) and protease activity (**B)** of 2 sets of single isolates (C1 and C2: Δ*lasR*Δ*pqsR*. C3 and C9: Δ*lasR*) from an evolved strain without microbiome presence (T18 L2 ctrl), selected depending on the presence and absence, respectively, of a mutation in *pqsR* (determined via Sanger sequencing). All isolates contained a mutation in *lasR*. Graphs show means, error bars indicate standard deviations. n ≥ 3, *p ≤ 0.05. (**C**) Photos of isolates C1, C2, C3, and C9 on LB agar showing the visible colour difference in the single isolates containing the different mutations.

Pyocyanin and protease production was quantified in two *lasR pqsR* (C1, C2) and two *lasR* mutant strains (C3, C9) isolated from the evolved control biofilm (T18 L2) in order to assess the role of *pqsR* in the production of these virulence factors. Both pyocyanin production and protease activity were significantly lower (p ≤ 0.05) in the isolates that contained both mutations (Fig. 4), consistent with previous reports of a regulatory role of PQS in pyocyanin and protease production, that are independent of LasR^62,63^.

### Evolved populations show a decreased susceptibility to β-lactam antibiotics

**Figure 5:**
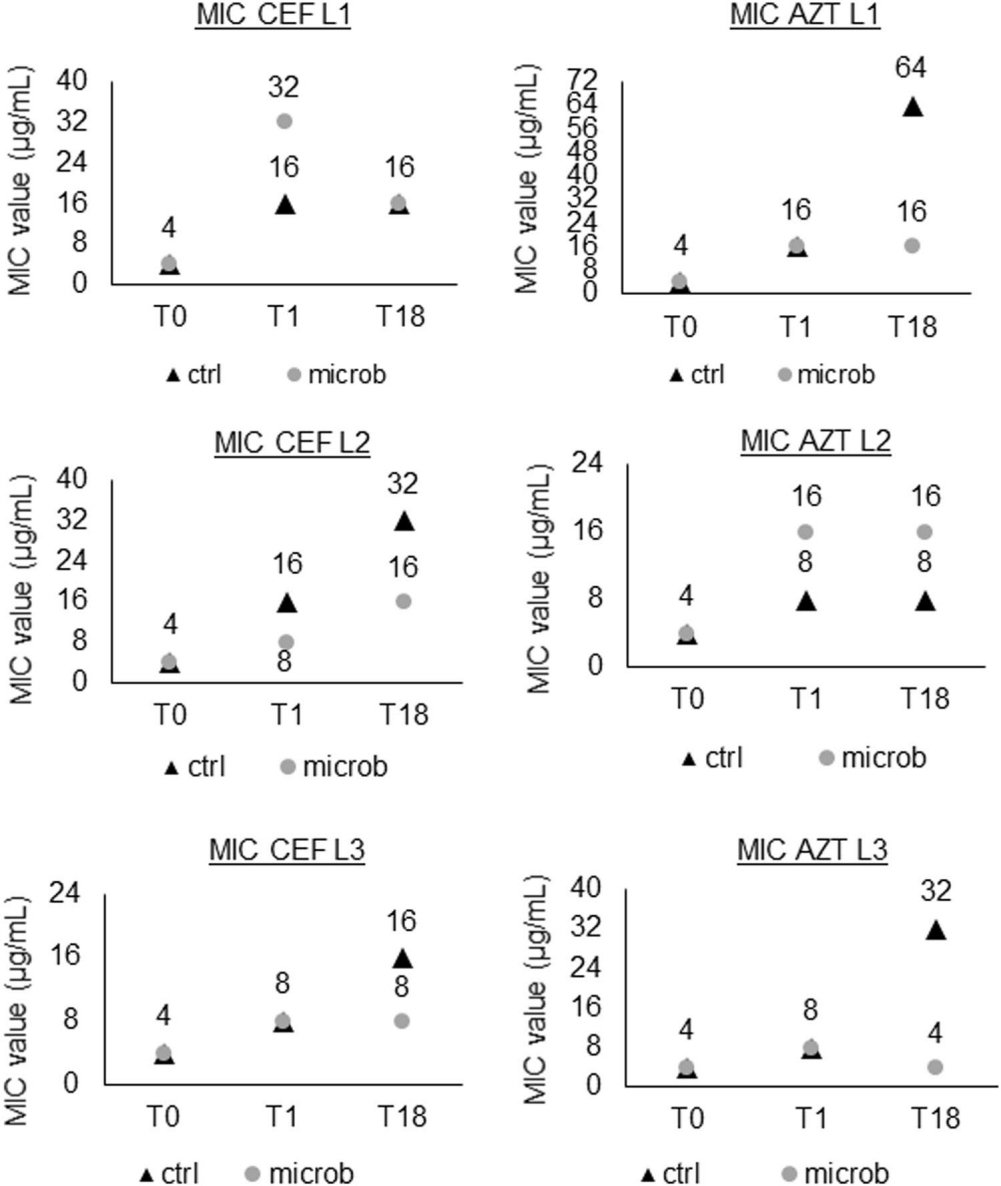
MIC values for ceftazidime (CEF) and aztreonam (AZT) in all lineages, for control (ctrl) and microbiome (microb) samples. T0: timepoint 0, planktonic start culture. T1: timepoint 1, 72 h biofilm cells. T18: timepoint 18, biofilm cells after 18 cycles (54 days). L1: lineage 1, L2: lineage 2, L3: lineage 3.

The influence of *P*. *aeruginosa* evolution in a biofilm in the presence or absence of microbiome members on antibiotic resistance was evaluated by testing the susceptibility of unevolved and evolved populations to four antibiotics commonly used to treat CF lung infections, each representing a different antibiotic class (Table S6). An increase in the MIC for ceftazidime and aztreonam was observed for evolved populations in all lineages, while no differences were noticed for ciprofloxacin, colistin, or tobramycin (Fig. 5).

The decreased susceptibility to ceftazidime and aztreonam mostly occurred early in the evolution study with an increase in MIC often already observed in T1 compared to T0. In two out of three lineages for both antibiotics, the decreased susceptibility was less pronounced in the presence of the microbiome.

### The competitive behaviour of evolved populations towards *S*. *aureus* is diminished

**Figure 6:**
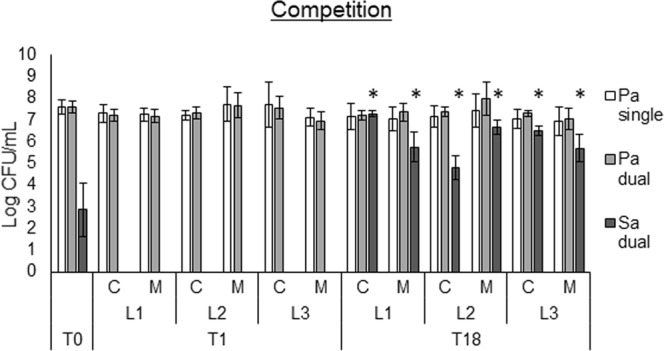
Determination of competition via dual-species biofilms of *P*. *aeruginosa* strains with *S*. *aureus* SP123. Pa single: *P*. *aeruginosa* strains as single-species biofilm, Pa dual: fraction of *P*. *aeruginosa* in the dual-species biofilm with *S*. *aure*us. Sa dual: fraction of *S*. *aureus* in the dual-species biofilm with *P*. *aeruginosa*. Graphs show means and error bars indicate standard deviations. n ≥ 3, *p ≤ 0.05 (*S. aureus* T18 versus T0 and T1). T0: timepoint 0, planktonic start culture. T1: timepoint 1, 72 h biofilm cells. T18: timepoint 18, biofilm cells after 18 cycles (54 days). L1: lineage 1, L2: lineage 2, L3: lineage 3. C: control single-culture. M: microbiome.

The fraction of *P*. *aeruginosa* and *S*. *aureus* present in 24 h dual-species biofilms was quantified on selective media. It was observed that in co-culture with evolved *P*. *aeruginosa* populations, *S*. *aureus* was significantly more present than with unevolved populations (T18 compared to T0 and T1) (Figs 6, S8P). Moreover, unevolved *P*. *aeruginosa* populations (T1) could outcompete *S*. *aureus* completely, showing no *S*. *aureus* growth in the biofilm, while *S*. *aureus* growth up to 7 log CFU/mL was observed in biofilms with the evolved populations.

## Discussion

In the present study, an early CF *P*. *aeruginosa* strain (AA2)^64^ was used to assess the evolution of biofilms in the absence or presence of the common CF lung microbiome members *S*. *aureus*, *S*. *anginosus*, *A*. *xylosoxidans*, *R*. *mucilaginosa*, and *G*. *haemolysans*. Since the CF lung microbiome is typically more diverse prior to *P*. *aeruginosa* colonization^65^, it is likely that early colonizers will co-exist with members of the microbiome, which could influence their evolution. Various phenotypic changes were observed after 18 evolution cycles, including decreased production of virulence factors (i.e. pyocyanin, protease, and rhamnolipids). These changes were mostly independent of the presence of other bacterial species and are similar to evolutionary changes observed for *P*. *aeruginosa* in chronic lung infections in CF patients.

Mutations were discovered in the genes *lasR* and *pqsR*, the regulators of two of the QS systems of *P*. *aeruginosa*^66^. The mutations in LasR were already present in the starting culture, and their frequency increased to nearly 100% in all lineages as early as cycle 1. The *in vitro* and *in vivo* loss of function of LasR has been described before in adapted *P*. *aeruginosa* strains both in planktonic and biofilm cultures^6,8,19,23–26^, and early genotypic and phenotypic changes during experimental evolution of *P*. *aeruginosa* have been reported as well^67,68^. Mutations in *lasR* only occurred after 30 days of biofilm culture in synthetic CF medium for strain PAO1^68^, which is in contrast with the results obtained in the present study for a CF clinical isolate. The presence of mutations in *lasR* at the start of the evolution study for the CF isolate used in the present study is presumably at the origin of differences with the study by Azimi *et al*.

Despite LasR probably being non-functional at T1, QS-regulated virulence factors (incl. proteases and pyocyanin), and 3-O-C_12_-HSL were still produced by these cultures. In stationary phase, *lasR* mutants in clinical isolate PA14 have been shown to activate QS regulated phenotypes by the *rhl* QS system replacing the *las* QS system and restoring the production of HSL, virulence factors, and *Pseudomonas* quinolone signal (PQS)^69^. It was also shown (for PAO1) that PQS production was delayed, but not abolished in a *lasR* mutant^70^. Furthermore, recent studies described a rewiring of the QS system in CF clinical isolates of *P*. *aeruginosa*, where RhlR can activate quorum-sensing-dependent genes independently of LasR^19^.

A subpopulation of *P*. *aeruginosa* developed mutations in *pqsR*, in addition to the mutations in *lasR*, at later stages of evolution. Mutations in *pqsR* have not been reported as part of the frequent adaptations in the CF lung, but have previously been observed *in vitro*^67,71^. Mutations in *pqsR* were associated with a pronounced effect on pyocyanin and protease production in our study, which is consistent with a previously described but unresolved mechanism by which PQS can activate transcription of these genes in a LasR-independent manner^62,63^.

PilA, the major pilin of the type IV pilus, was mutated in all lineages, with or without microbiome. Type IV pili are involved in biofilm formation in *P*. *aeruginosa*, they mediate attachment to surfaces and surface motility^72^. Klausen *et al*.^73^ demonstrated (in strain PAO1) that in a *pilA*/wt mixed biofilm the mutant was only present in the stalks of the mushrooms-shaped structures, whereas the wild type formed the caps. Furthermore, a *pilApqsA* double mutant in *P*. *aeruginosa* PAO1 only formed surface attached microcolonies and no mushroom-shaped structures^74^. Interestingly, in our study the mutations in *pqsR* and in *pilA* seemed to develop in parallel.

The *pilA* mutation reported in this study is a deletion of a 6 nt duplicated sequence, leading to the deletion of two threonine residues out of stretch of four threonines located at the C-terminal of this protein, 3 amino acids upstream of the stop codon. PilA in *P*. *aeruginosa* AA2 has only 41% similarity to PilA in PAO1, it is more similar to PilA in *P*. *aeruginosa* K122-4, a clinical isolate from a CF patient in Toronto^75^. The stretch of threonines in which the deletion occurs is not present in the PAO1 PilA. It is therefore unknown if the deletion in PilA of strain AA2 has an effect on biofilm phenotype. Nonetheless, the slight decrease in biofilm formation of evolved populations (which was observed when pooling the data of all lineages), as well as the low frequency of mutations in flagellar genes and SagS point to a modulation of attachment and biofilm formation in evolved populations. While the ability of the evolved population to form new biofilms was decreased (probably due to the described genotypic changes), the biofilm formation during evolution increased over time. We speculate that this could be due to phenotypic alterations, such as accumulation of extracellular matrix components during transfers when starting new biofilm cycles.

A decrease in susceptibility to the β-lactam antibiotics ceftazidime and aztreonam was observed for all evolved *P*. *aeruginosa* populations. Evolution of antibiotic resistance in response to continuous antibiotic stress is common^76,77^. However, in our study set-up, no antibiotics were present. Davies *et al*.^78^ and D’Argenio *et al*.^8^ also observed this increase in MIC for ceftazidime with evolved *P*. *aeruginosa* strains in the absence of antibiotics. This was attributed to an elevated production of β-lactamases by QS mutant strains. Although no mutations were found in either *ampC* or *poxB*, the described co-regulation between AmpR, a β-lactamase expression regulator, and LasR could explain the observed increase in resistance^8,79,80^. Additionally, in the WGS data, a mutation was observed in the gene encoding MexL, a TetR family repressor of the multidrug efflux pump MexJK. This mutation was more frequently observed in lineages that evolved in the absence of the microbiome, and could possibly explain the more pronounced increase in the MIC of β-lactam antibiotics under these conditions. Nevertheless, MexJK has only been shown to transport tetracycline, erythromycin, and triclosan in *P*. *aeruginosa*^60,61^, and the potential transport of β-lactam antibiotics by the MexJK efflux pump remains to be investigated.

Finally, the competitive behaviour of *P*. *aeruginosa* in a dual-species biofilm with *S*. *aureus* was investigated, showing that unevolved populations outcompete *S*. *aureus*, while evolved populations allowed ample *S*. *aureus* biofilm growth. Co-cultures of *P*. *aeruginosa* and *S*. *aureus* have been studied extensively and mostly show *P*. *aeruginosa* outcompeting *S*. *aureus in vitro* (reviewed in^81^). Yet, *in vivo*, concomitant infections of both pathogens are often seen^82^. Here, we show that evolutionary adaptation of *P*. *aeruginosa* leads to a less competitive phenotype. These results can most likely be explained by the observed mutations in *lasR* and *pqsR* and downstream effects on the production of QS molecules and virulence factors (such as proteases, pyocyanin), which have been shown to affect *S*. *aureus* growth and/or viability^81^. For example, mutations in *P*. *aeruginosa* leading to impaired 2-hydroxy-4-alkylquinolone production – which is regulated by the PQS QS system - reduced the growth inhibition of *S*. *aureus*, normally inflicted by the wild-type strains^83,84^.

In a recent evolution study by Tognon *et al*.^24^, *P*. *aeruginosa* and *S*. *aureus* were co-cultured as dual-species biofilms, but a *lasR* mutation was only observed in monocultures of *P*. *aeruginosa*, and not in co-cultured samples. In our study, *lasR* mutations increased in frequency regardless of whether other species were present. However, in our model the community was more diverse and no direct interactions occurred between different species, in contrast to previous studies. As the spatial organization of complex communities influences metabolism and co-evolution of multispecies communities^85^, this could explain differences between the present study and previous work^24^. However, the lack of direct contact may be more reflective of the *in vivo* situation in the CF lungs, as bacteria are often found as separate single species microcolonies in sputum of CF patients, rather than as truly intermixed communities^4,86^.

In the present study, we performed phenotypic and genotypic analysis of the whole evolved population. With this approach, possible effects of evolution on population diversification in the presence or absence of the microbiome were not considered. Phenotypic diversity in the CF lung environment is frequently observed for *P*. *aeruginosa* and other CF pathogens^5,87^, which is possibly related to regional adaptation^18^. *In vitro* evolution studies have also reported *P*. *aeruginosa* diversification during biofilm growth, with variation in antibiotic resistance and colony morphology being observed^78,88^. Whether interspecies interactions between the members of the microbiome could influence *P*. *aeruginosa* population diversity remains to be determined.

In conclusion, we showed that *in vitro* evolution of *P*. *aeruginosa* AA2 biofilms can lead to pathoadaptive mutations and phenotypes commonly found in *in vivo* chronic infections. The most noticeable genotypic and phenotypic changes, which are related to the *las* and *pqs* QS machineries, occurred regardless of the presence of other members of the CF microbiome. Understanding the *in vivo* factors that drive and/or modulate the evolutionary behaviour of *P*. *aeruginosa* may lead to novel therapeutic avenues to direct evolution towards a genotype/phenotype that is susceptible to treatment.

## Supplementary information

Supplemental figures and tables

## Data Availability

All data is available in this manuscript, the Supplemental Information or deposited in databases, as described in the manuscript.
