# Supplementary material for: In vitro evolution of Pseudomonas aeruginosa AA2 biofilms in the presence of cystic fibrosis lung microbiome members
Source: Sci Rep. 2019 Sep 6;9:12859. doi: 10.1038/s41598-019-49371-y (PMC6731285; doi:10.1038/s41598-019-49371-y)
Supplement: Supplementary file 1 — Supplemental figures and tables [file 41598_2019_49371_MOESM1_ESM.pdf]

Supplementary data

In vitro evolution of *Pseudomonas aeruginosa* AA2 biofilms in the presence of cystic fibrosis lung microbiome members

Eva Vandeplassche [a], Andrea Sass [a], Astrid Lemarcq [a], Ajai A. Dandekar [b], Tom Coenye [a], and Aurélie Crabbé [a]

[a] Laboratory of Pharmaceutical Microbiology, Ghent University, Belgium
[b] Department of Medicine/Department of Microbiology, University of Washington, USA

Supplemental figure legends

Figure S1. Composition of the microbiome biofilm community in the evolution model (in the presence of *P. aeruginosa* AA2) after 24, 48 and 72 hours. Sa: *S. aureus*, Sag: *S. anginosus*, Ax: *A. xylosoxidans*, Rm: *R. mucilaginosa*, Gh: *G. haemolysans.* Strains were plated on selective media: LB agar with 7.5% NaCl, LB agar with 0.31 mg/L triclosan, McConkey agar with 5 mg/L aztreonam, nutrient agar with 5 mg/L mupirocin and 5 mg/L colistin sulphate, and Columbia agar base with 32/6.4 mg/L co-trimoxazole (respectively). [34] Graph shows mean log CFU/mL and error bars indicate standard deviations, n = 3.

Figure S2. Biofilm formation of *P. aeruginosa* AA2 determined directly at each timepoint during the evolution study (T1-T18) for lineages 1, 2 and 3 (L1, L2 and L3). Log CFU/mL of *P. aeruginosa* control (ctrl ο) or in the presence of the microbiome (microb Δ) was quantified via the plating method. T1-T18: timepoint 1 to timepoint 18 (72 hour cycles).

Figure S3. Biofilm formation in LB (aerobic conditions) for each evolved culture (T1, T18) and the planktonic start culture (T0) was determined (24 h) via **(A)** CFU plating and **(B)** crystal violet staining (biomass) of strains in phenotypic experiments after -80°C preservation. Graphs show means and error bars indicate standard deviations. n ≥ 3, * p ≤ 0.05.

Figure S4. Swimming (A), swarming (B), and twitching (C) motility of T0, T1, and T18 *P. aeruginosa* strains. Graphs show means, error bars indicate standard deviations, n ≥ 3. T0: timepoint 0, planktonic culture. T1: timepoint 1, 72h biofilm cells. T18: timepoint 18, biofilm cells after 18 cycles (54 days). L1: lineage 1, L2: lineage 2, L3: lineage 3. C: control single-culture, M: microbiome.

Figure S5. 24 hour planktonic growth curves for lineage 1, 2 and 3 (L1, L2 and L3). T0 (□) and T1 (control □; microbiome □) strains show a significant difference in absorbance in the stationary phase compared to T18 (control ●; microbiome▲) strains. Graphs show means, error bars indicate standard deviations. n = 3. T0: timepoint 0, planktonic culture. T1: timepoint 1, 72h biofilm cells. T18: timepoint 18, biofilm cells after 18 cycles (54 days).

Figure S6. Determination of CFU/mL after 24 h growth. C = control; M = microbiome. Graphs show means, error bars indicate standard deviations. n = 3. T0: timepoint 0, planktonic culture. T1: timepoint 1, 72h biofilm cells. T18: timepoint 18, biofilm cells after 18 cycles (54 days). L1: lineage 1, L2: lineage 2, L3: lineage 3. C: control single-culture, M: microbiome.

Figure S7. Quantification of the NF-κB pathway activation (A) and cytotoxicity (B) induced by the (un-) evolved *P. aeruginosa* strains after 4h (inflammation) or 6h (viability) of infection in the 3-D A549 lung epithelial model. Graphs show means, error bars indicate standard deviations. n = 3, no significant differences. Neg. ctrl: negative control of uninfected cells. T0: timepoint 0, planktonic culture. T1: timepoint 1, 72h biofilm cells. T18: timepoint 18, biofilm cells after 18 cycles (54 days). L1: lineage 1, L2: lineage 2, L3: lineage 3. C: control single-culture, M: microbiome.

Figure S8. Phenotypic data presented as average of three independent lineages (pooled).

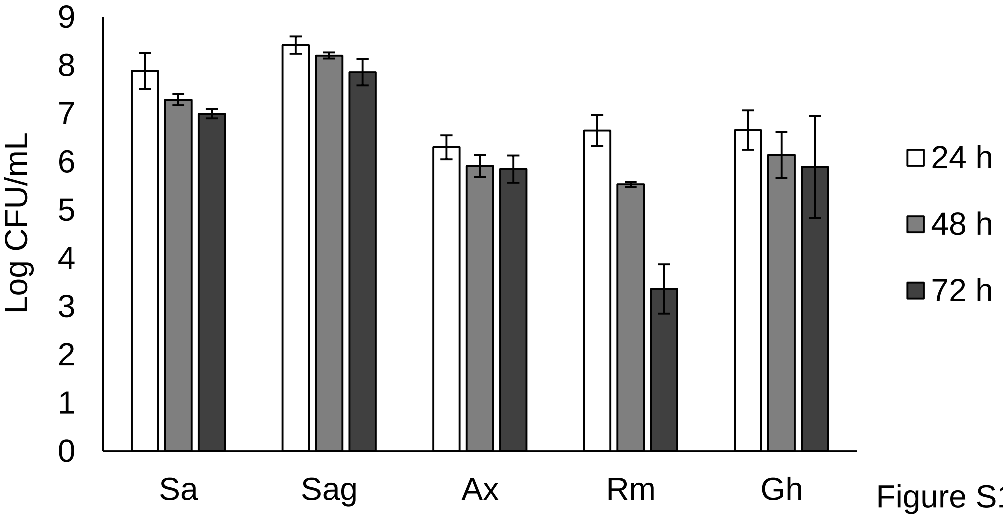

Figure S1

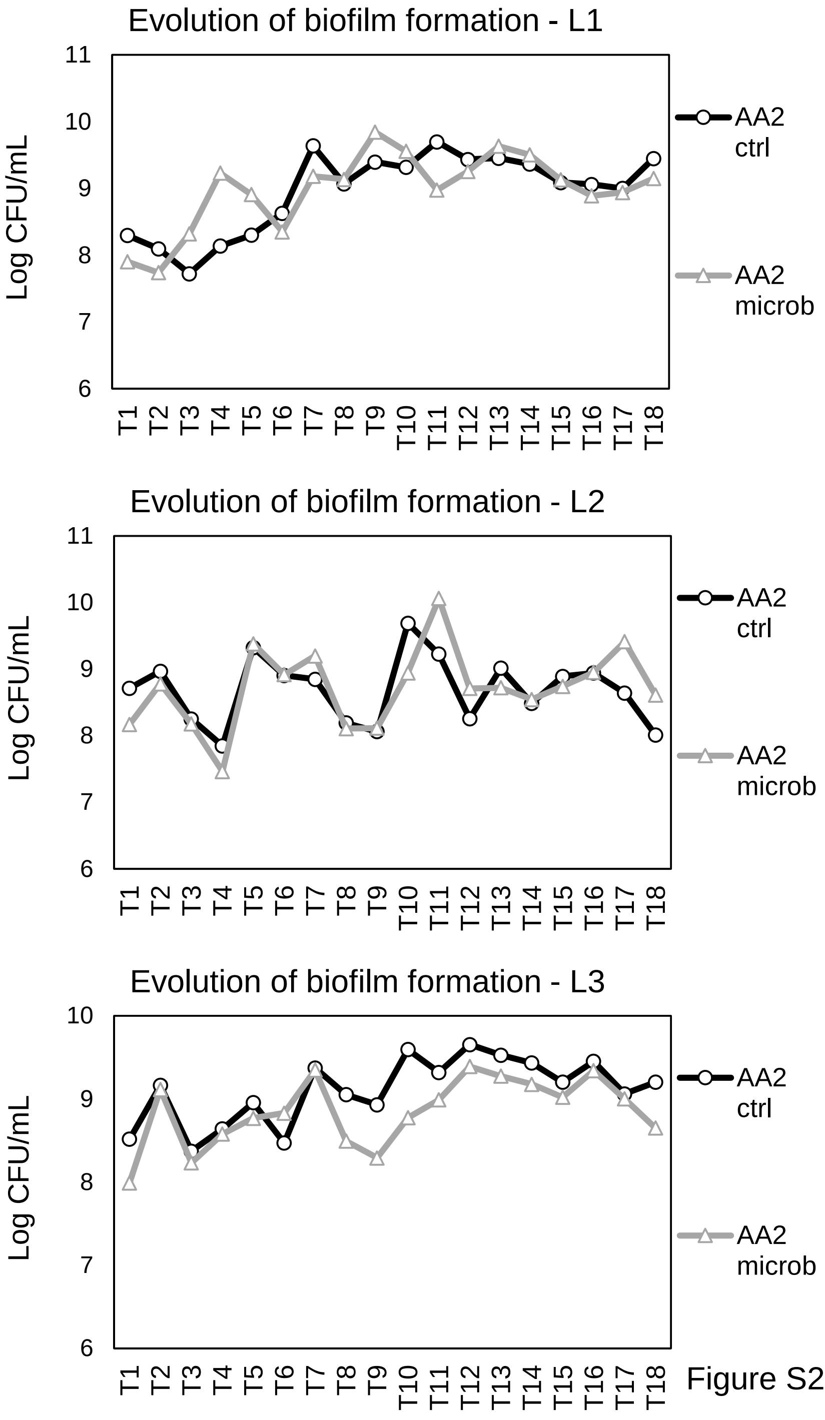
Evolution of biofilm formation - L1
Log CFU/mL
6
7
8
9
10
11
T1 T2 T3 T4 T5 T6 T7 T8 T9 T10 T11 T12 T13 T14 T15 T16 T17 T18
AA2 ctrl
AA2 microb
Evolution of biofilm formation - L2
Log CFU/mL
6
7
8
9
10
11
T1 T2 T3 T4 T5 T6 T7 T8 T9 T10 T11 T12 T13 T14 T15 T16 T17 T18
AA2 ctrl
AA2 microb
Evolution of biofilm formation - L3
Log CFU/mL
6
7
8
9
10
T1 T2 T3 T4 T5 T6 T7 T8 T9 T10 T11 T12 T13 T14 T15 T16 T17 T18
AA2 ctrl
AA2 microb

Figure S2

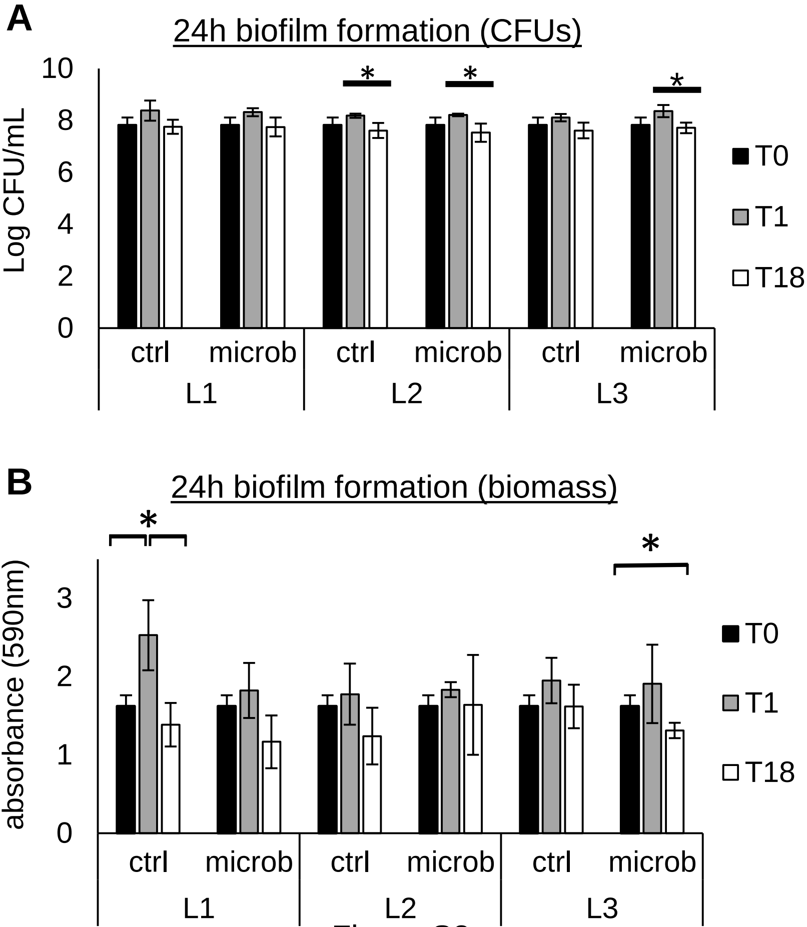
A
24h biofilm formation (CFUs)
Log CFU/mL
10
8
6
4
2
0
*
*
*
ctrl
microb
ctrl
microb
ctrl
microb
L1
L2
L3
T0
T1
T18
B
24h biofilm formation (biomass)
absorbance (590nm)
3
2
1
0
*
*
ctrl
microb
ctrl
microb
ctrl
microb
L1
L2
L3
T0
T1
T18

Figure S3

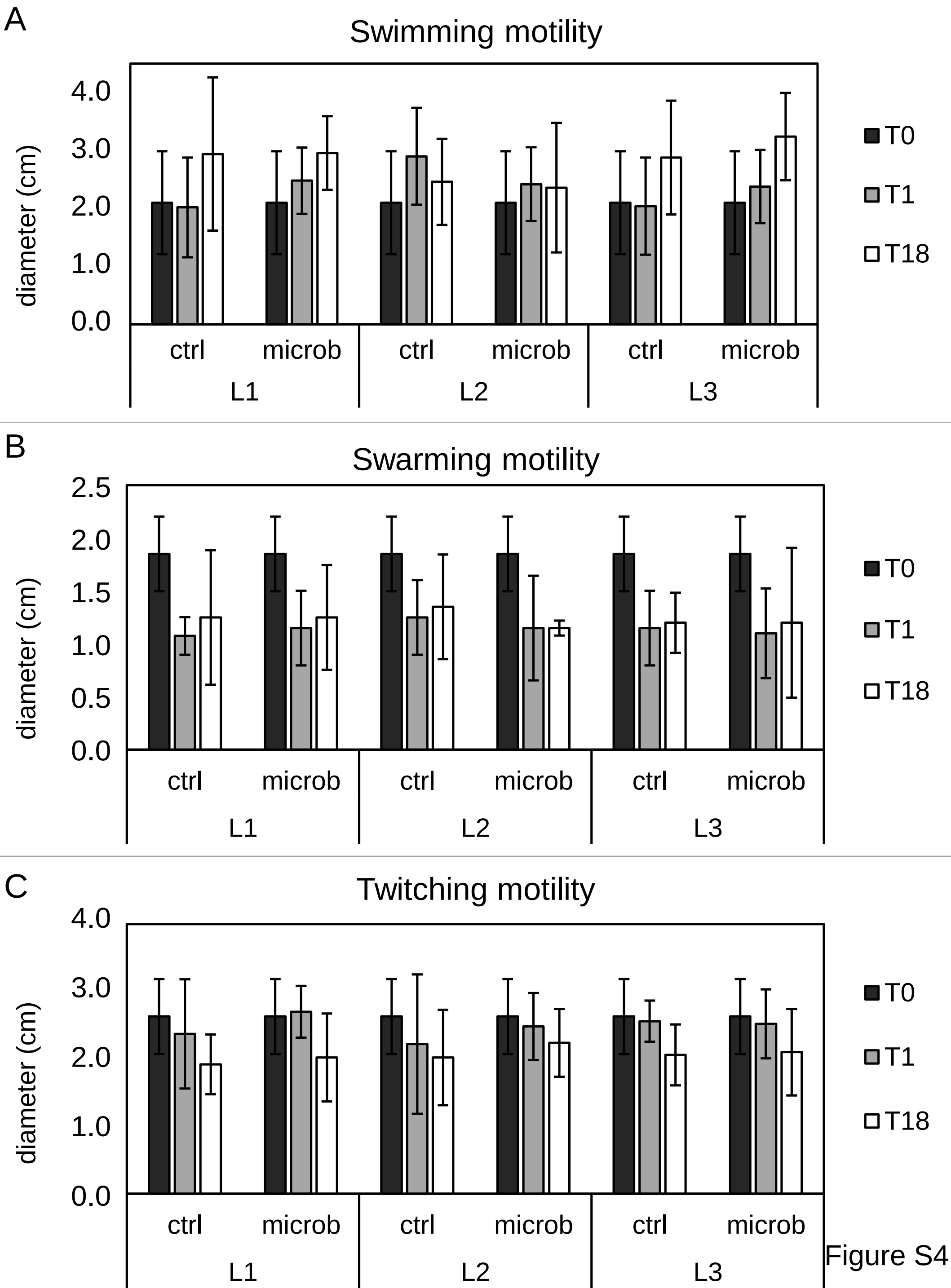

A
Swimming motility
diameter (cm)
4.0
3.0
2.0
1.0
0.0
ctrl
microb
L1
L2
L3
T0
T1
T18
B
Swarming motility
2.5
2.0
1.5
1.0
0.5
0.0
C
Twitching motility

Figure S4

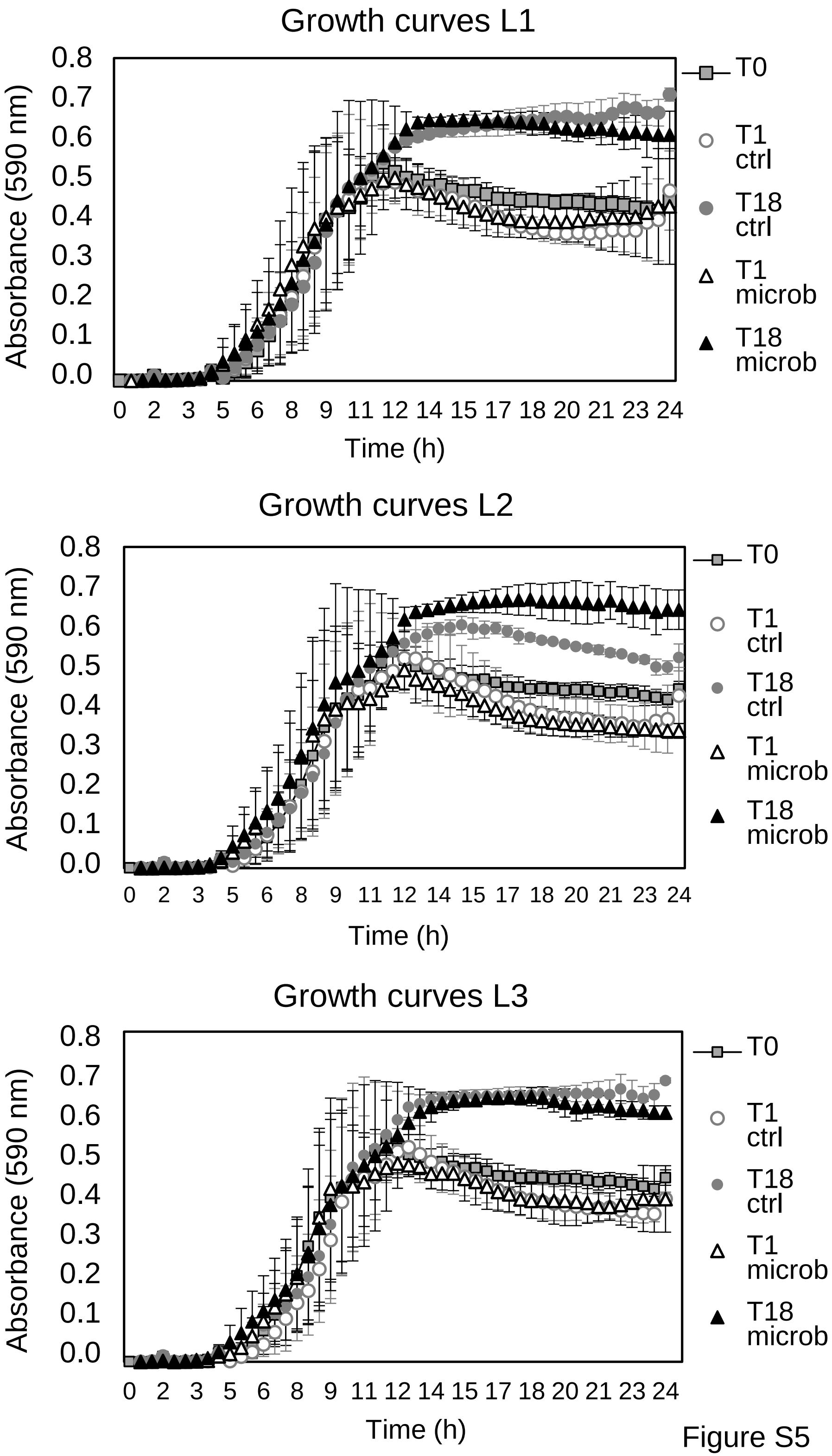
Growth curves L1
Absorbance (590 nm)
0.0 0.1 0.2 0.3 0.4 0.5 0.6 0.7 0.8
0 2 3 5 6 8 9 11 12 14 15 17 18 20 21 23 24
Time (h)
T0
T1 ctrl
T18 ctrl
T1 microb
T18 microb
Growth curves L2
Absorbance (590 nm)
0.0 0.1 0.2 0.3 0.4 0.5 0.6 0.7 0.8
0 2 3 5 6 8 9 11 12 14 15 17 18 20 21 23 24
Time (h)
T0
T1 ctrl
T18 ctrl
T1 microb
T18 microb
Growth curves L3
Absorbance (590 nm)
0.0 0.1 0.2 0.3 0.4 0.5 0.6 0.7 0.8
0 2 3 5 6 8 9 11 12 14 15 17 18 20 21 23 24
Time (h)
T0
T1 ctrl
T18 ctrl
T1 microb
T18 microb

Figure S5

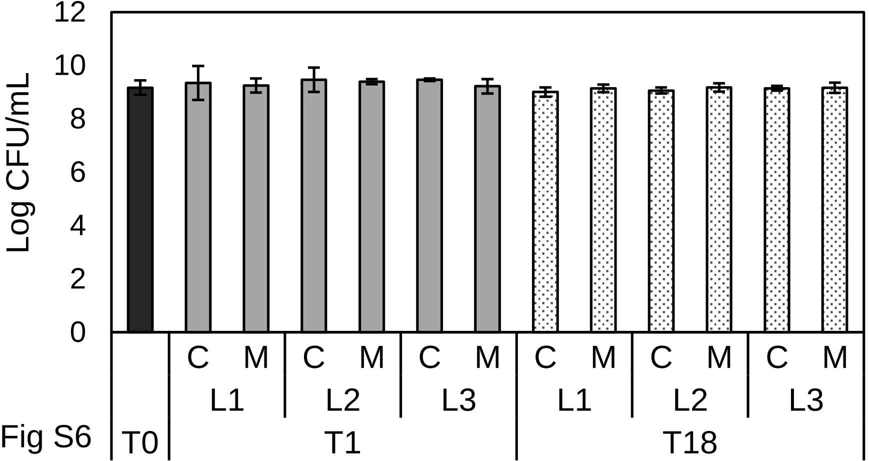

Fig S6

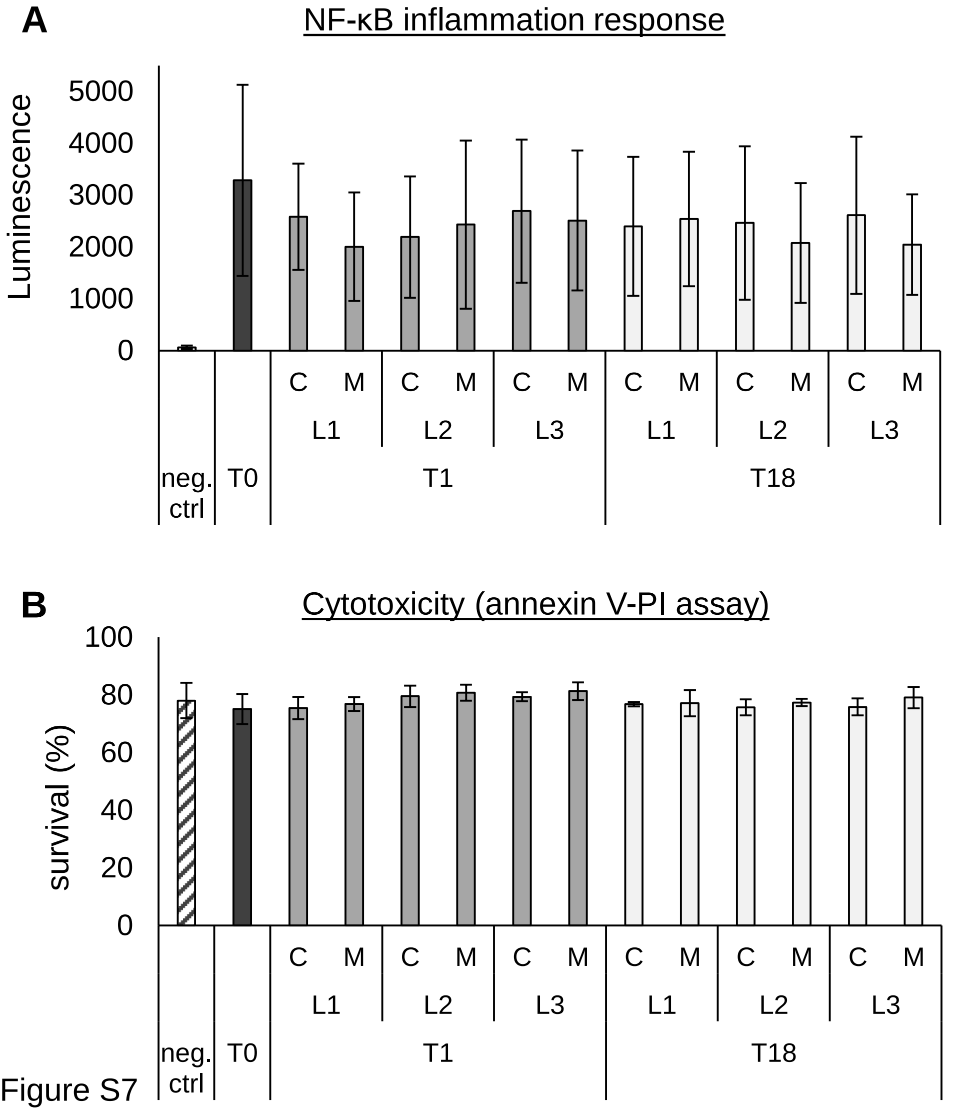
A
NF-κB inflammation response
Luminescence
5000
4000
3000
2000
1000
0
C
M
L1
L2
L3
neg. ctrl
T0
T1
T18
B
Cytotoxicity (annexin V-PI assay)
survival (%)
100
80
60
40
20
0

Figure S7

Fig. S8 A Evolution of biofilm formation - POOLED

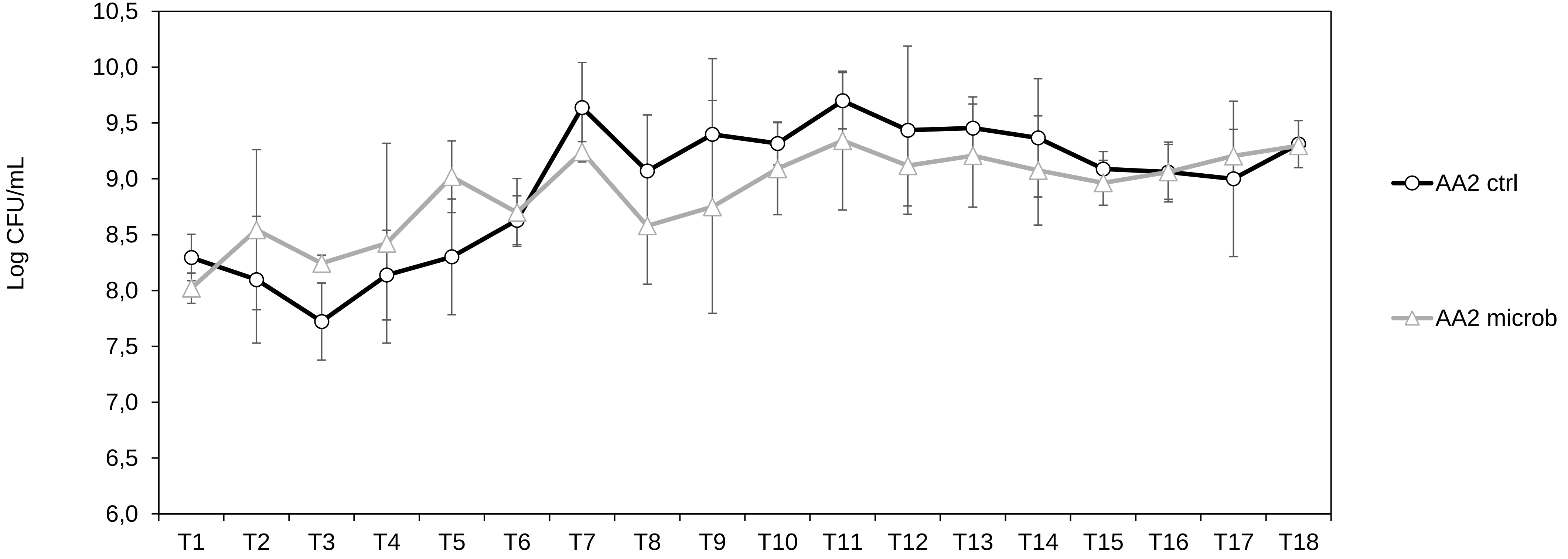

Biofilm formation of *P. aeruginosa* AA2 determined directly at each timepoint during the evolution study (T1-T18). Graphs show means of three independent lineages (L1, L2, L3), error bars indicate standard deviations**.** Log CFU/mL of *P. aeruginosa* control (ctrl ο) or in the presence of the microbiome (microb Δ) was quantified via the plating method. T1-T18: timepoint 1 to timepoint 18 (72 hour cycles).

Fig S8 B 24h biofilm formation of *P. aeruginosa* evolution samples - POOLED

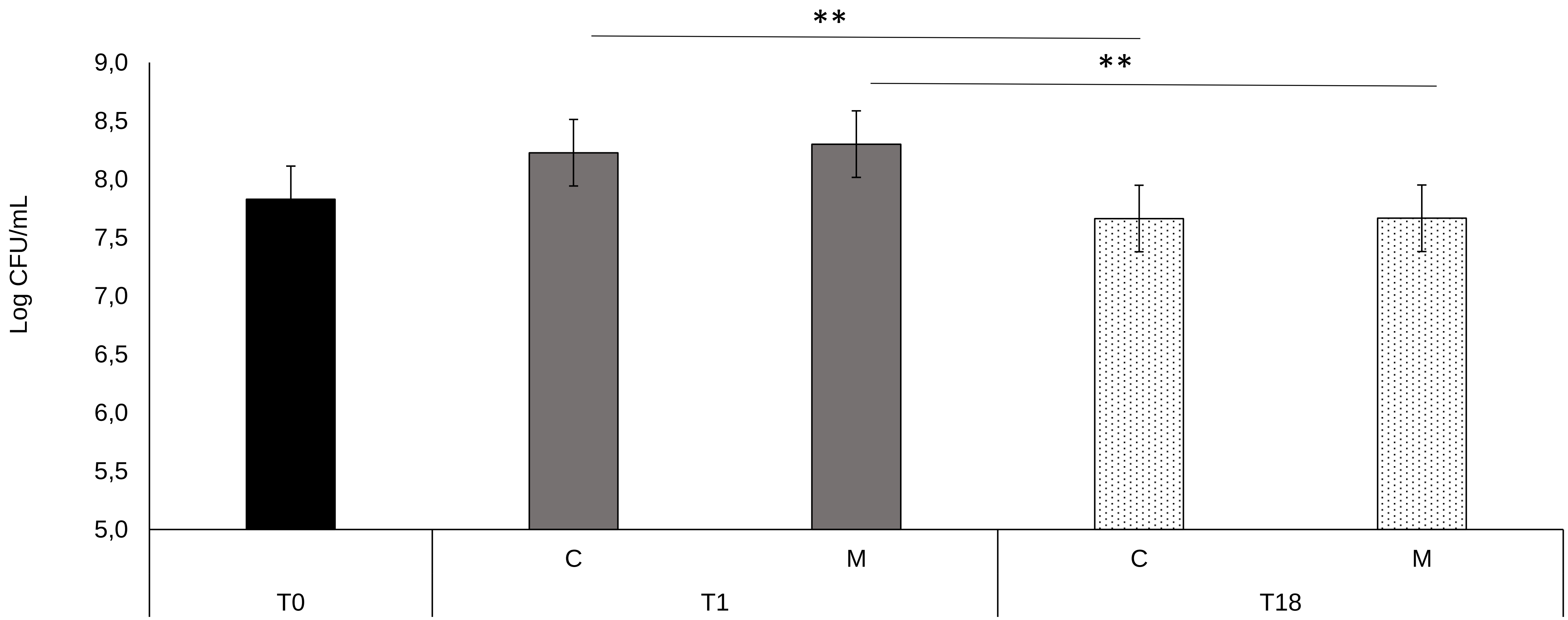

Biofilm formation in LB (aerobic conditions) for evolved cultures (T1, T18) and the planktonic start culture (T0) was determined (24 h) via CFU plating after -80°C preservation. Graphs show means of three independent lineages (L1, L2, L3) and error bars indicate standard deviations. ** $p \leq 0.01$.

Fig S8 C Biofilm formation (biomass) - POOLED

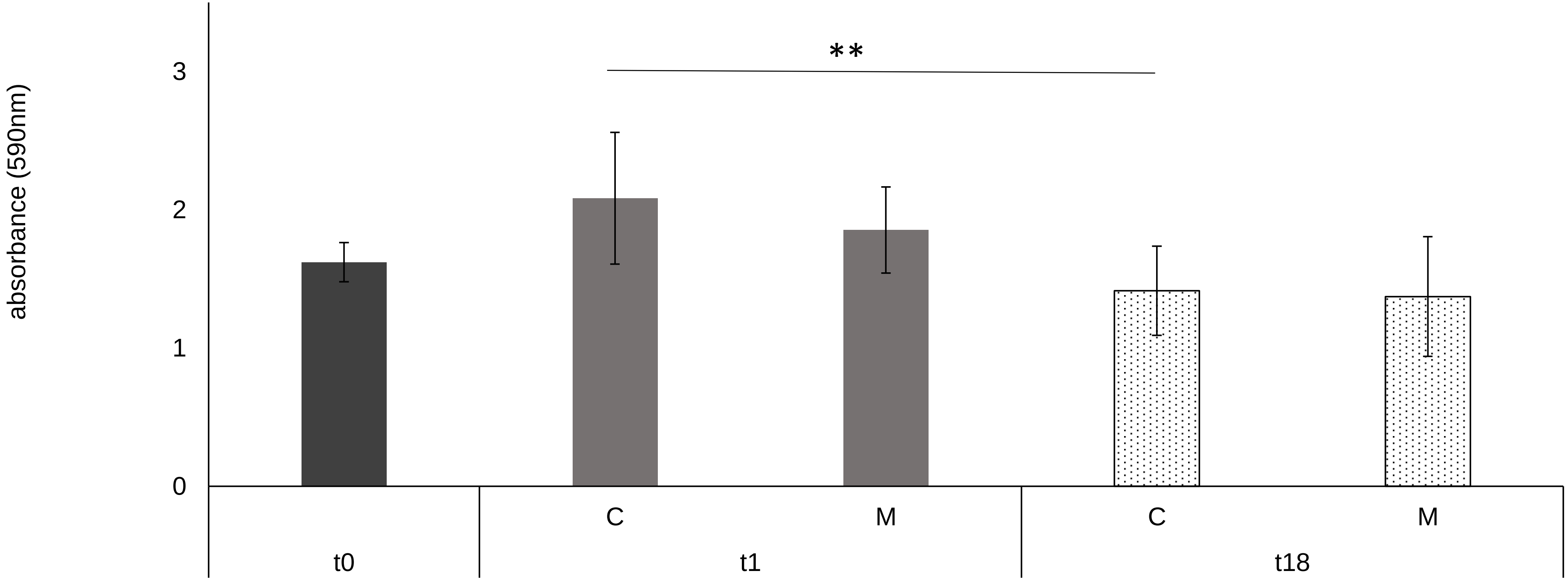

Biofilm formation in LB (aerobic conditions) for evolved cultures (T1, T18) and the planktonic start culture (T0) was determined (24 h) via crystal violet staining (biomass) after -80°C preservation. Graphs show means of three independent lineages (L1, L2, L3) and error bars indicate standard deviations. ** $p \leq 0.01$.

Fig S8 D

3-O-C_{12}-HSL quantification - POOLED

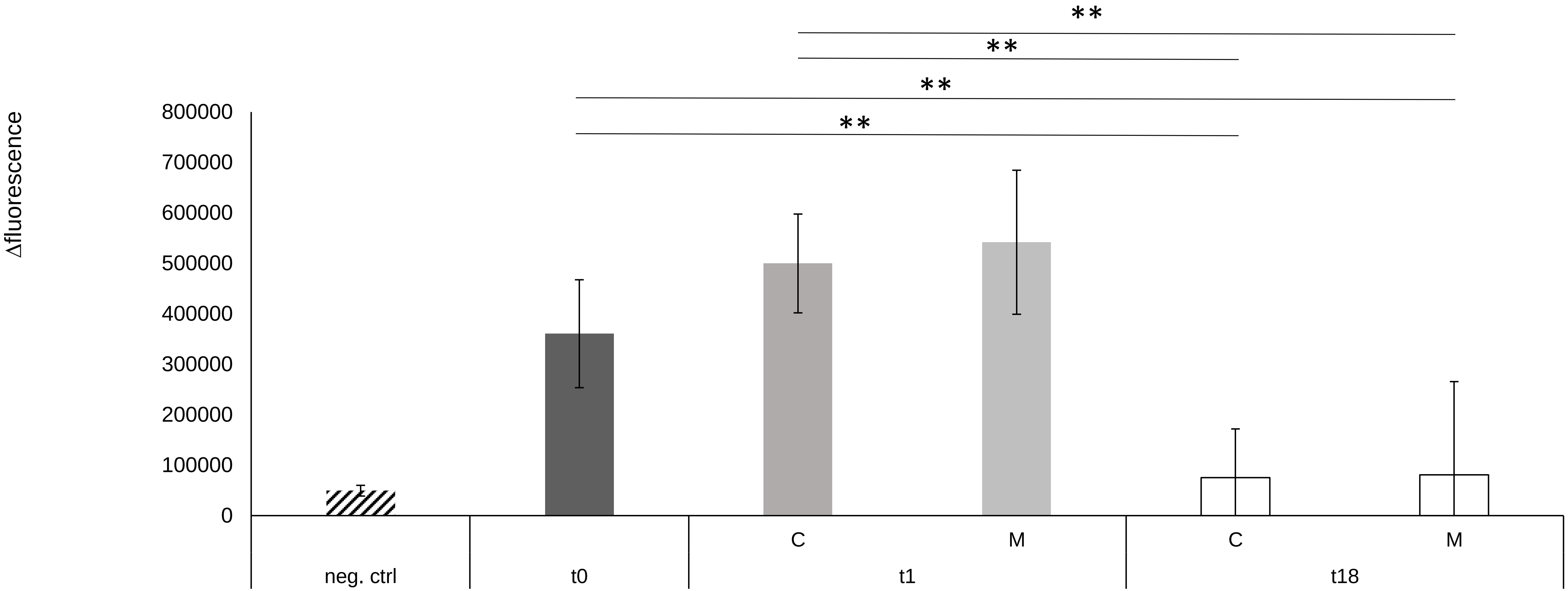

Quantification of 3-O-C_{12}-HSL production using an *E. coli* biosensor strain (expressing GFP in response to this molecule). Graphs show means of three independent lineages (L1, L2, L3), error bars indicate standard deviations, * $p \leq 0.05$, ** $p \leq 0.01$. T0: timepoint 0, planktonic culture. T1: timepoint 1, 72h biofilm cells. T18: timepoint 18, biofilm cells after 18 cycles (54 days). C: control single-culture, M: microbiome. Neg. ctrl: negative control, pure *E. coli* culture.

Fig S8 E Pyocyanin - POOLED

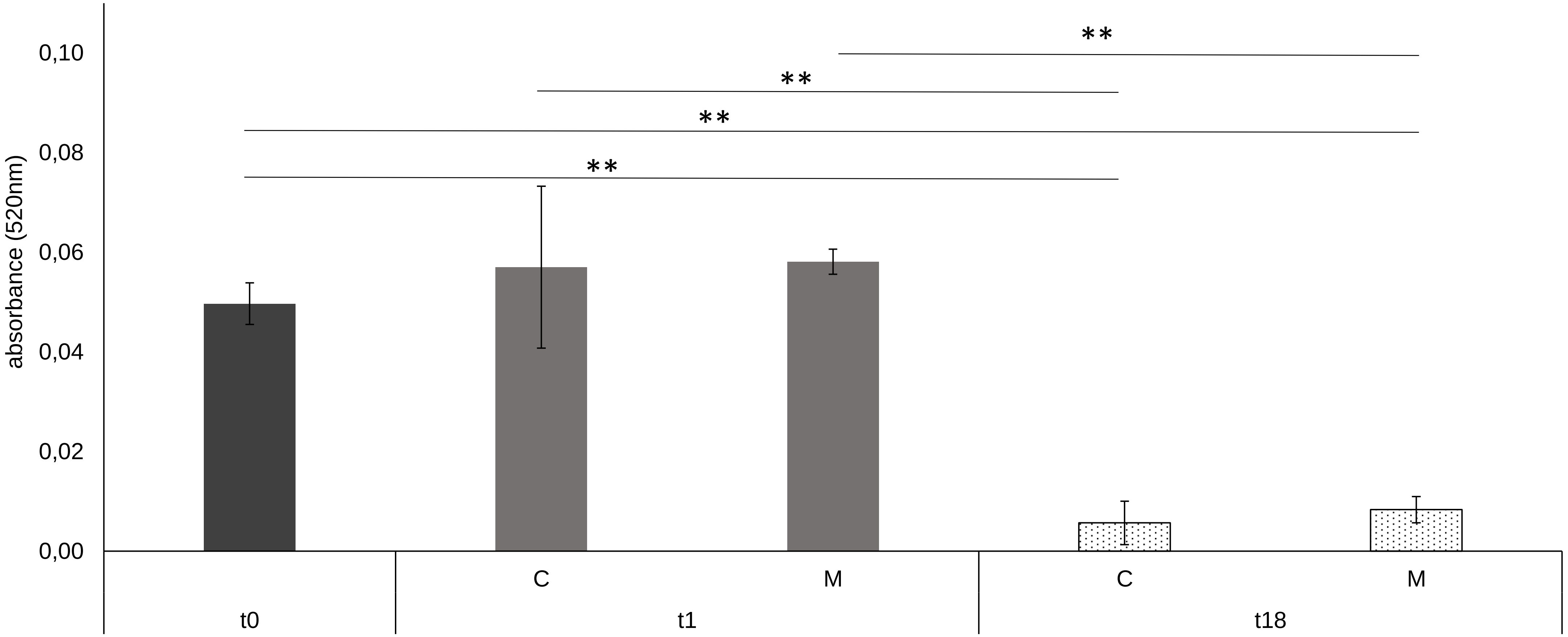

Determination of pyocyanin production by unevolved and evolved *P. aeruginosa* AA2 strains. Graphs show means of three independent lineages (L1, L2, L3), error bars indicate standard deviations, ** $p \leq 0.01$. T0: timepoint 0, planktonic culture. T1: timepoint 1, 72h biofilm cells. T18: timepoint 18, biofilm cells after 18 cycles (54 days). C: control single-culture, M: microbiome.

Fig S8 F Protease (azocasein assay) - POOLED

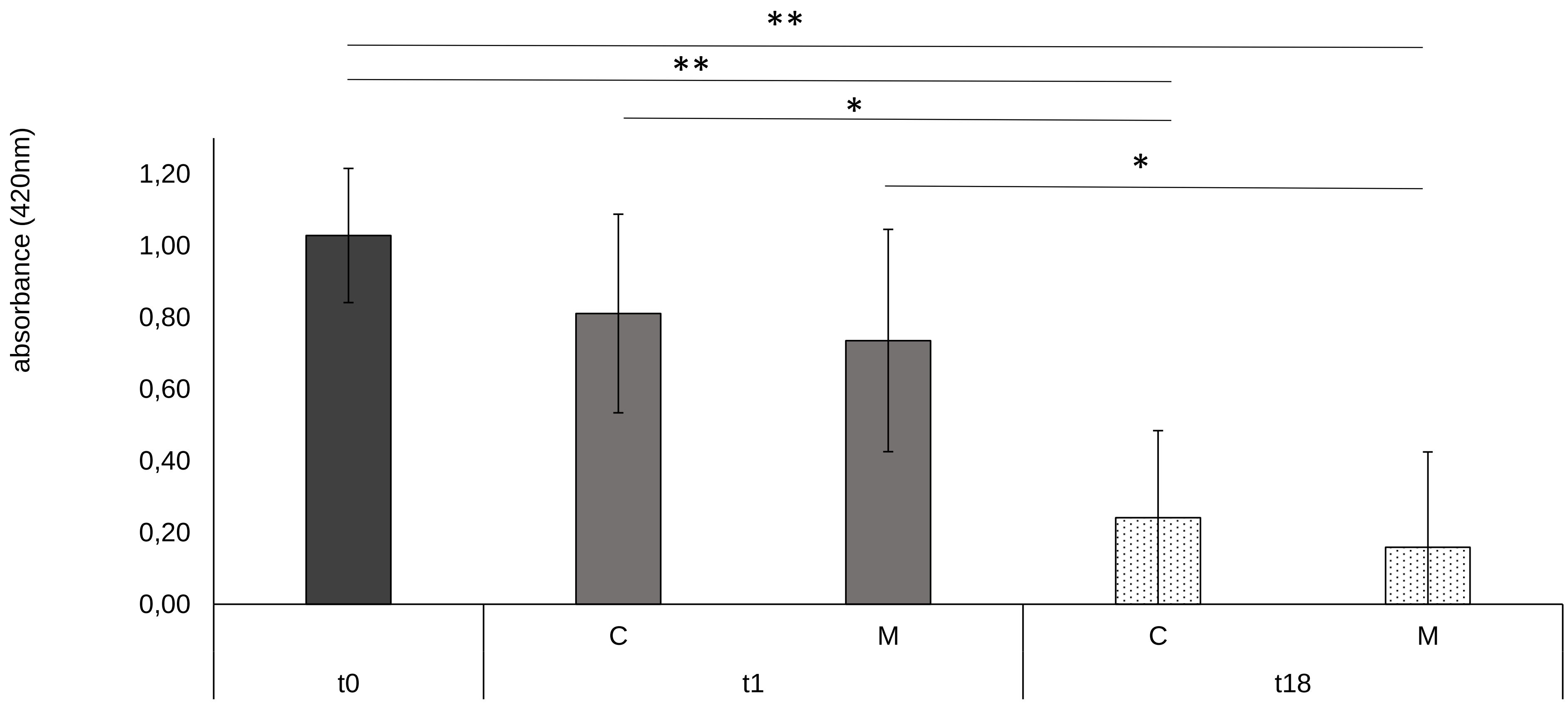

Determination of protease production by unevolved and evolved *P. aeruginosa* AA2 strains. Graphs show means of three independent lineages (L1, L2, L3), error bars indicate standard deviations, * $p \leq 0.05$, ** $p \leq 0.01$. T0: timepoint 0, planktonic culture. T1: timepoint 1, 72h biofilm cells. T18: timepoint 18, biofilm cells after 18 cycles (54 days). C: control single-culture, M: microbiome.

Fig S8 G Rhamnolipids -POOLED

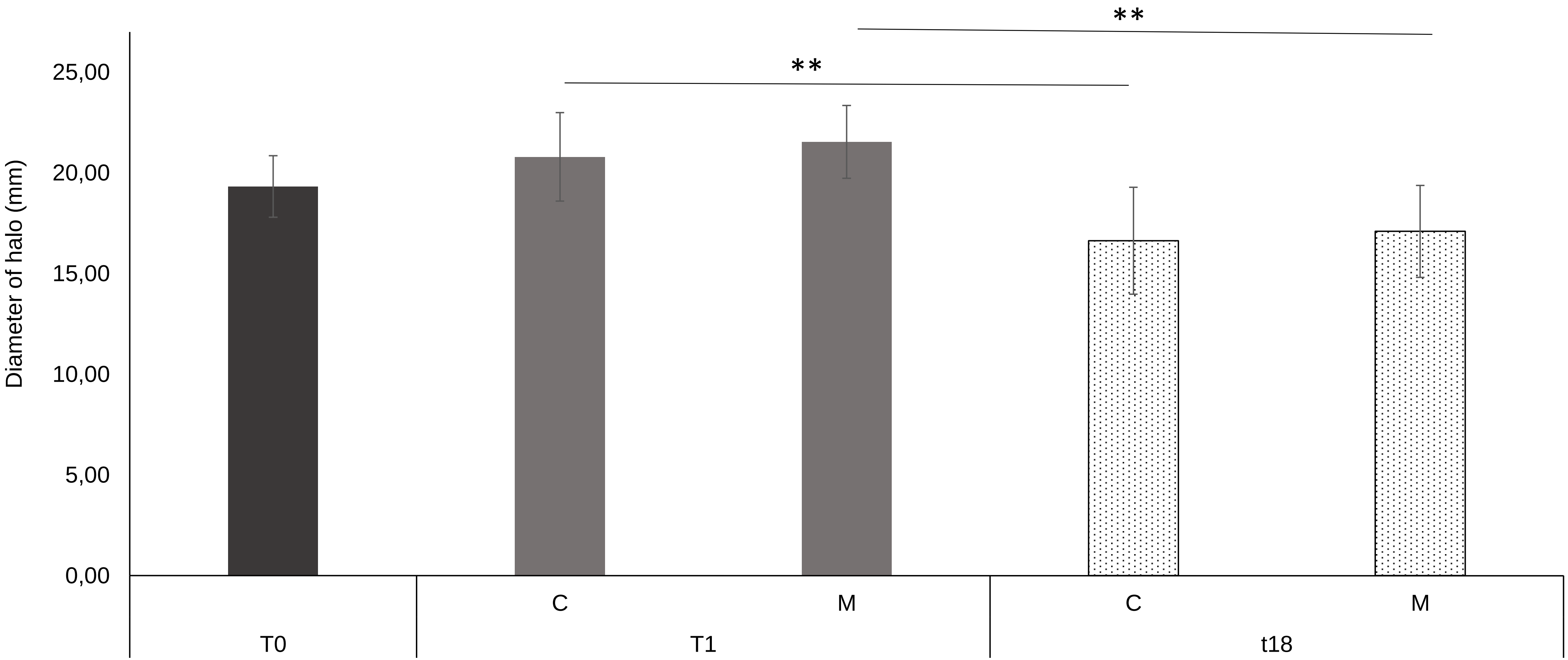

Determination of rhamnolipids production by unevolved and evolved *P. aeruginosa* AA2 strains. Graphs show means of three independent lineages (L1, L2, L3), error bars indicate standard deviations, ** $p \leq 0.01$. T0: timepoint 0, planktonic culture. T1: timepoint 1, 72h biofilm cells. T18: timepoint 18, biofilm cells after 18 cycles (54 days). C: control single-culture, M: microbiome.

Fig S8 H Pyoverdine - POOLED

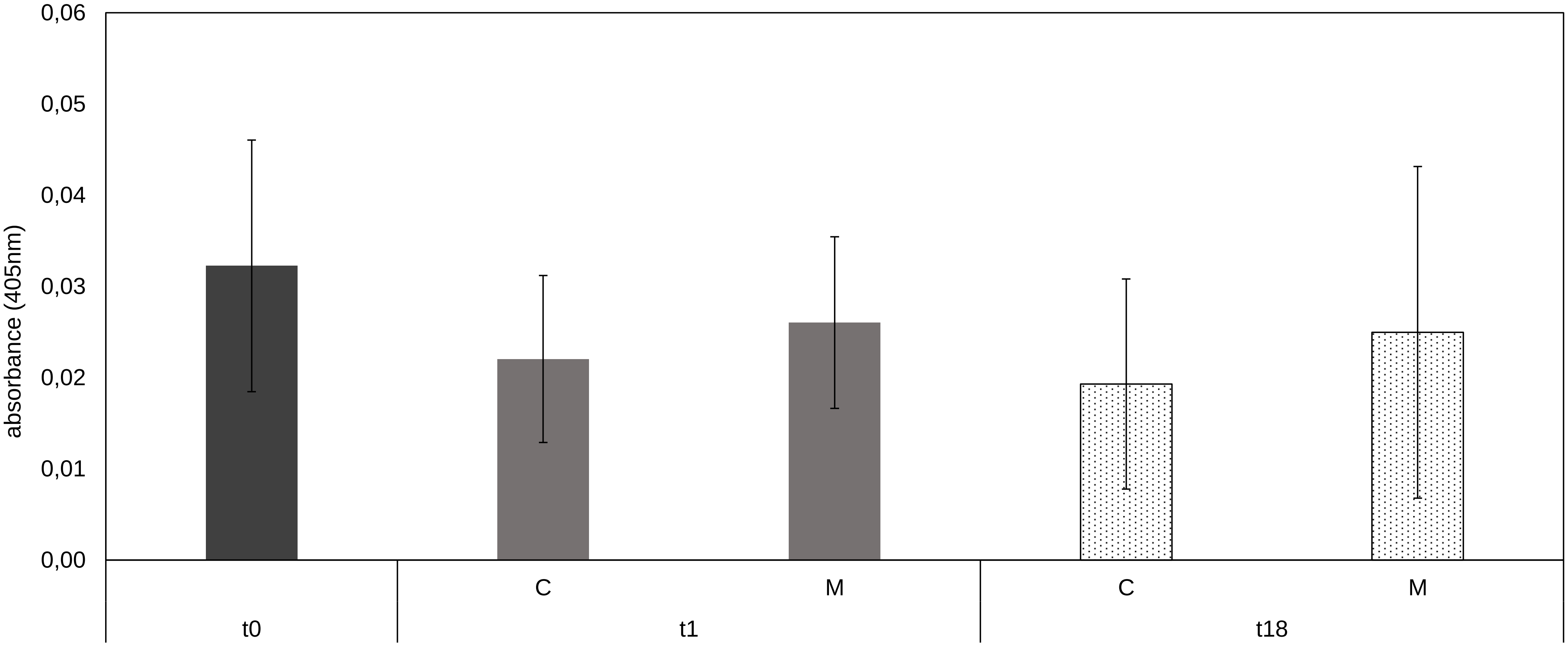

Determination of pyoverdin production by unevolved and evolved *P. aeruginosa* AA2 strains. Graphs show means of three independent lineages (L1, L2, L3), error bars indicate standard deviations. T0: timepoint 0, planktonic culture. T1: timepoint 1, 72h biofilm cells. T18: timepoint 18, biofilm cells after 18 cycles (54 days). C: control single-culture, M: microbiome.

Fig S8 I Swimming motility - POOLED

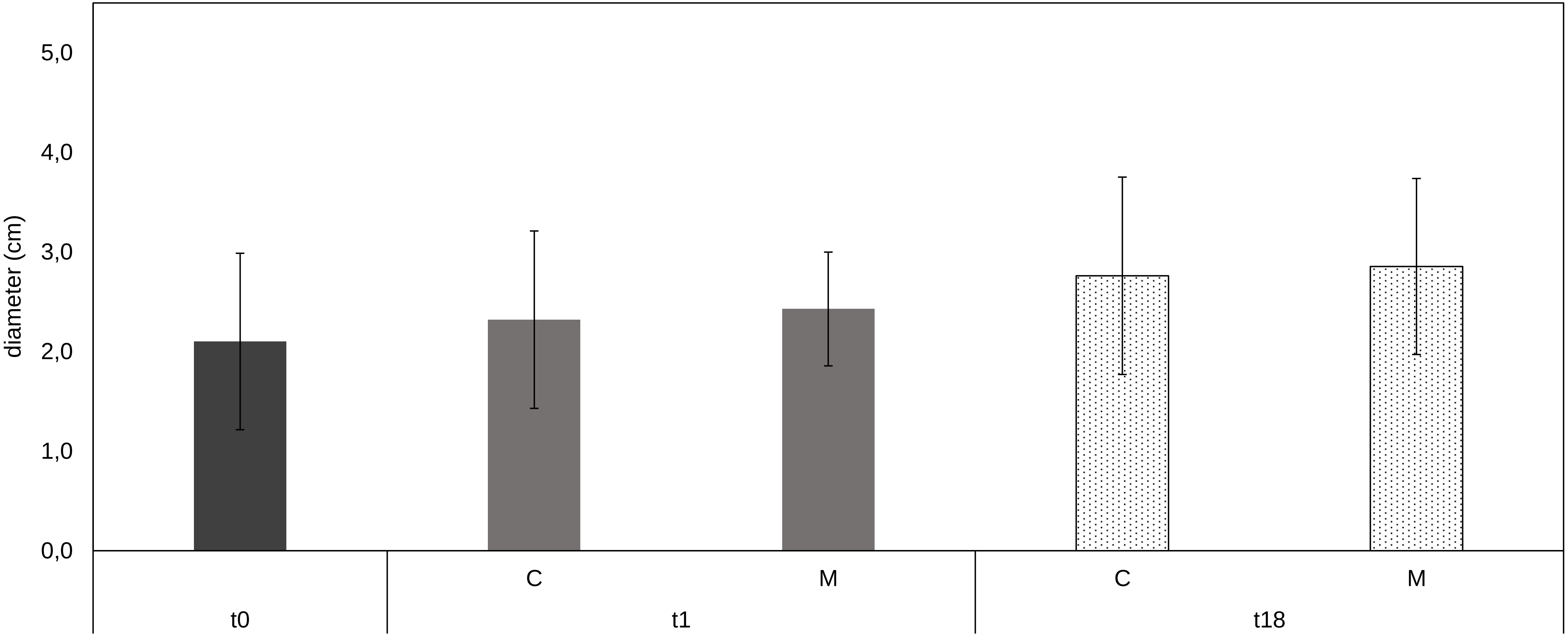

Swimming motility of T0, T1, and T18 *P. aeruginosa* strains. Graphs show means of three independent lineages (L1, L2, L3), error bars indicate standard deviations. T0: timepoint 0, planktonic culture. T1: timepoint 1, 72h biofilm cells. T18: timepoint 18, biofilm cells after 18 cycles (54 days). C: control single-culture, M: microbiome.

Fig S8 J Swarming motility - POOLED

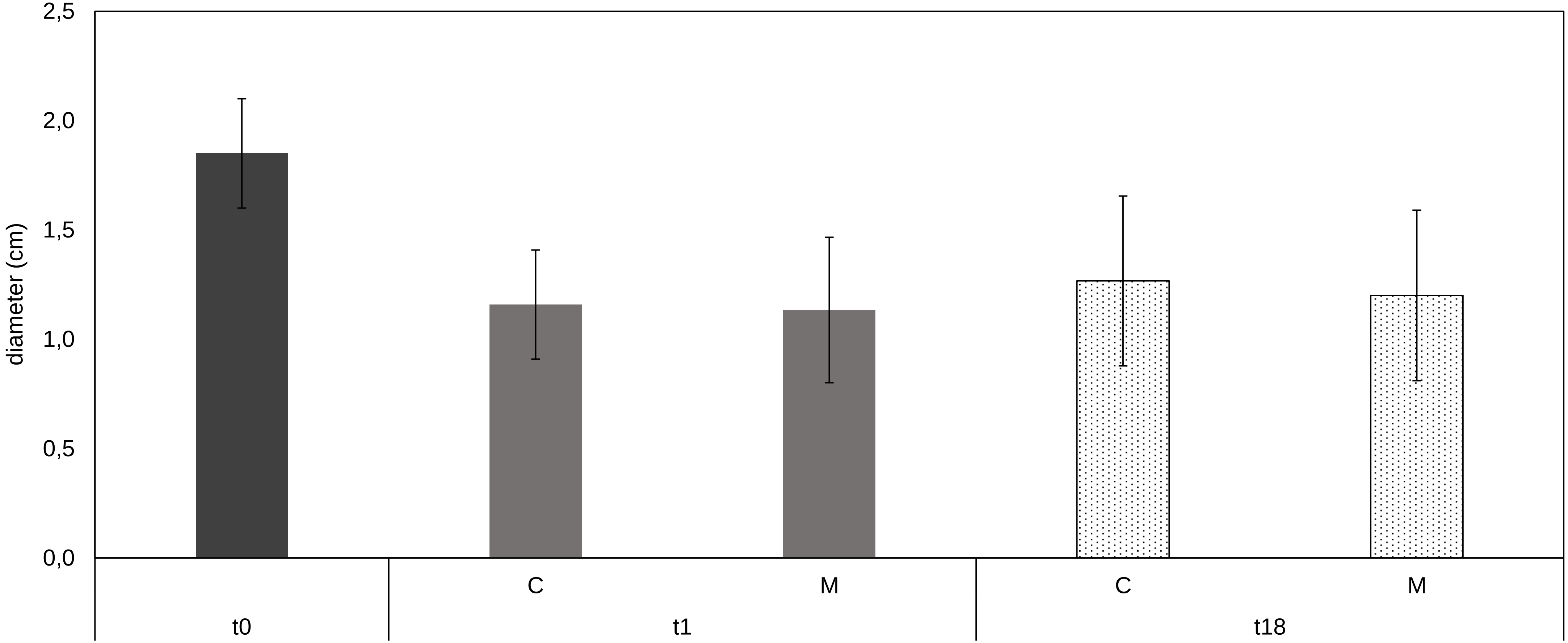

Swarming motility of T0, T1, and T18 *P. aeruginosa* strains. Graphs show means of three independent lineages (L1, L2, L3), error bars indicate standard deviations. T0: timepoint 0, planktonic culture. T1: timepoint 1, 72h biofilm cells. T18: timepoint 18, biofilm cells after 18 cycles (54 days). C: control single-culture, M: microbiome.

Fig S8 K Twitching motility - POOLED

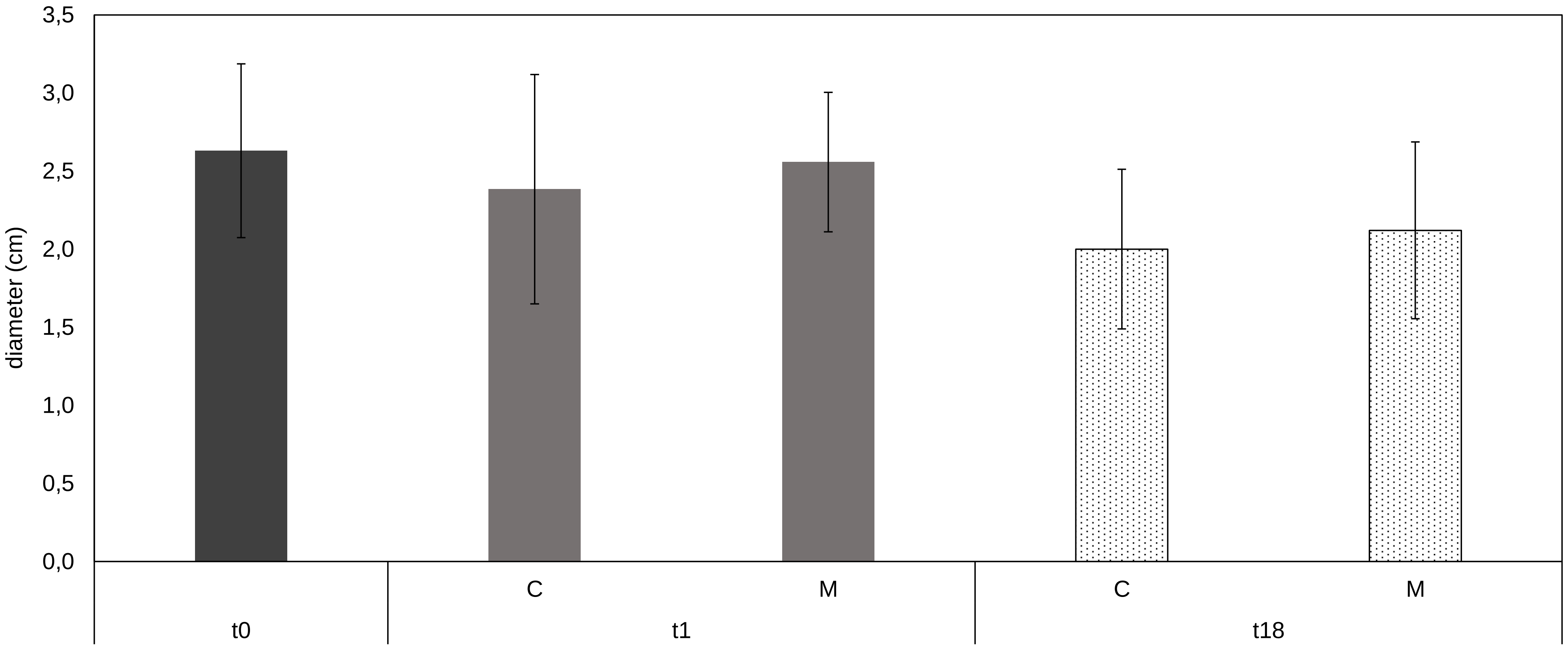

Twitching motility of T0, T1, and T18 *P. aeruginosa* strains. Graphs show means of three independent lineages (L1, L2, L3), error bars indicate standard deviations. T0: timepoint 0, planktonic culture. T1: timepoint 1, 72h biofilm cells. T18: timepoint 18, biofilm cells after 18 cycles (54 days). C: control single-culture, M: microbiome.

Fig S8 L Growth curves - POOLED

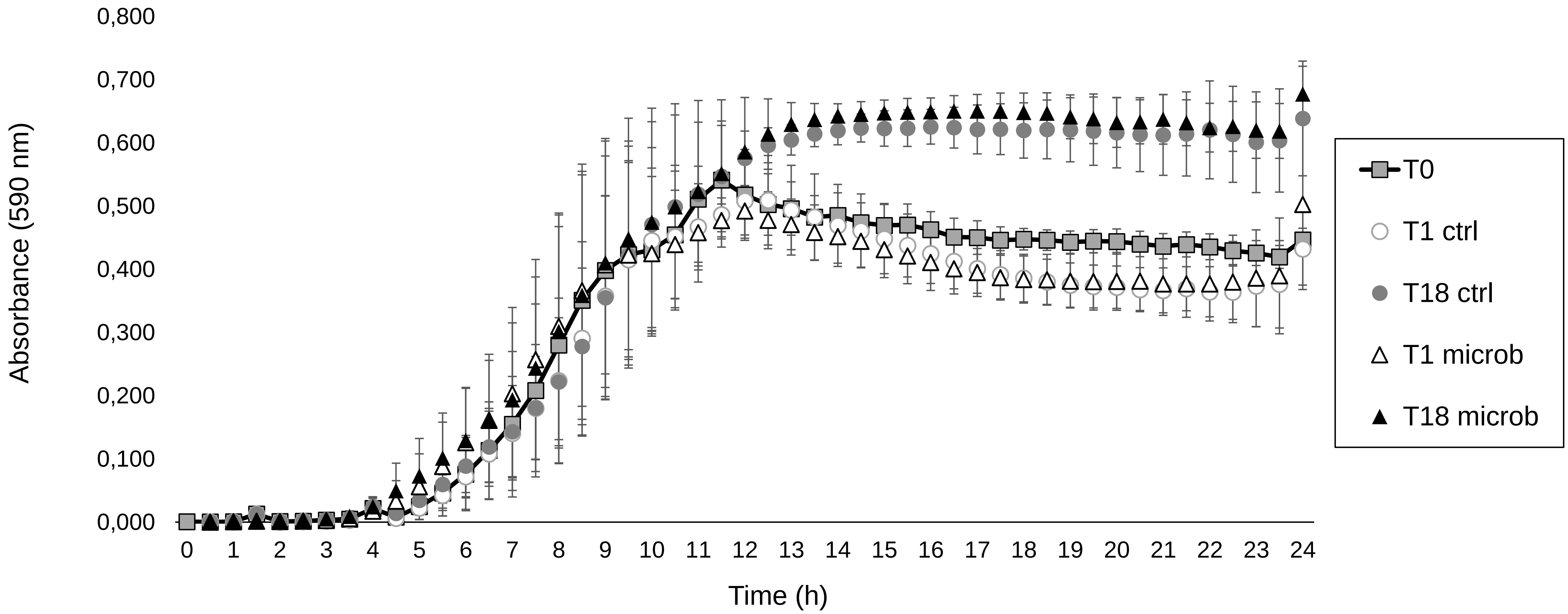

24 hour planktonic growth curves. T0 (□) and T1 (control □; microbiome □) strains show a significant difference in absorbance in the stationary phase compared to T18 (control ●; microbiome ▲) strains. Graphs show means, of three independent lineages (L1, L2, L3), error bars indicate standard deviations. n = 3. T0: timepoint 0, planktonic culture. T1: timepoint 1, 72h biofilm cells. T18: timepoint 18, biofilm cells after 18 cycles (54 days).

Fig S8 M CFU plating 24h growth curve - POOLED

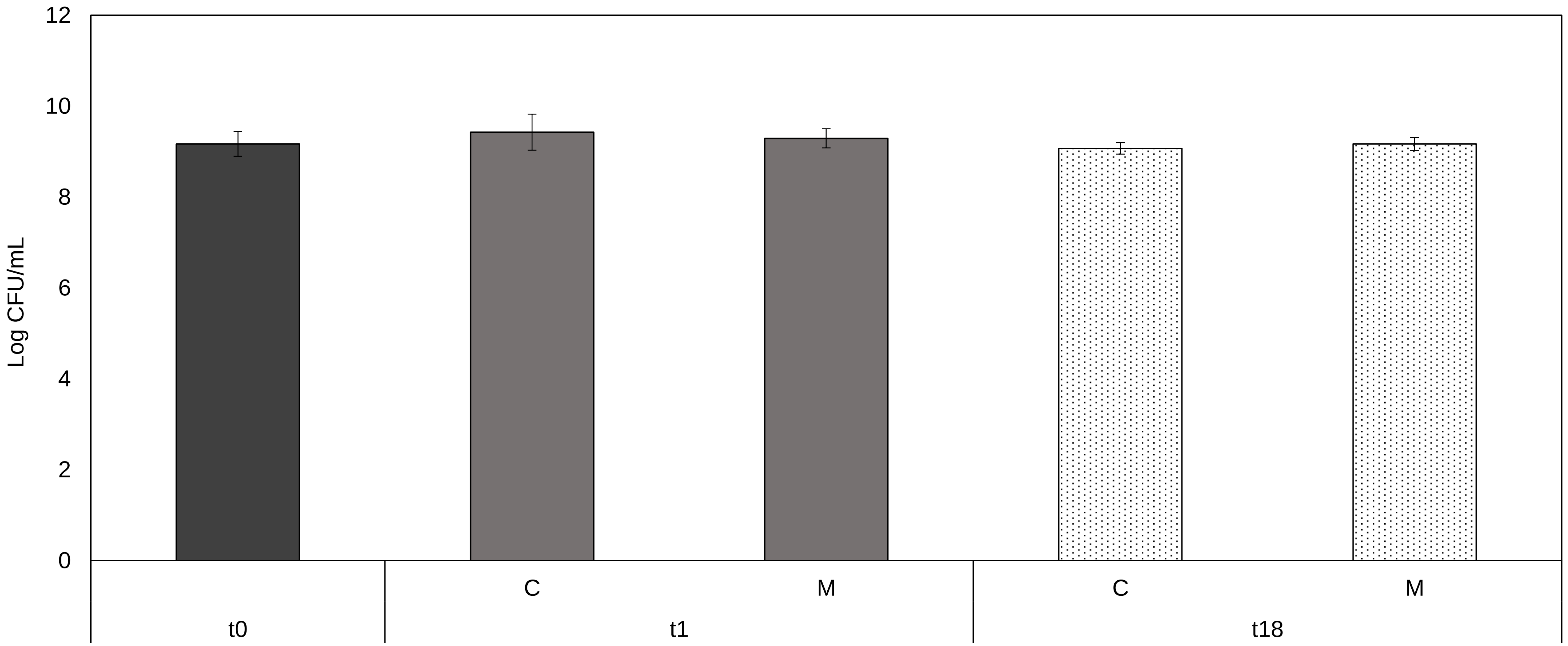

Determination of CFU/mL after 24 h growth. C = control; M = microbiome. Graphs show means of three independent lineages (L1, L2, L3), error bars indicate standard deviations. T0: timepoint 0, planktonic culture. T1: timepoint 1, 72h biofilm cells. T18: timepoint 18, biofilm cells after 18 cycles (54 days). C: control single-culture, M: microbiome.

Fig S8 N   NF-kB inflammation response - POOLED

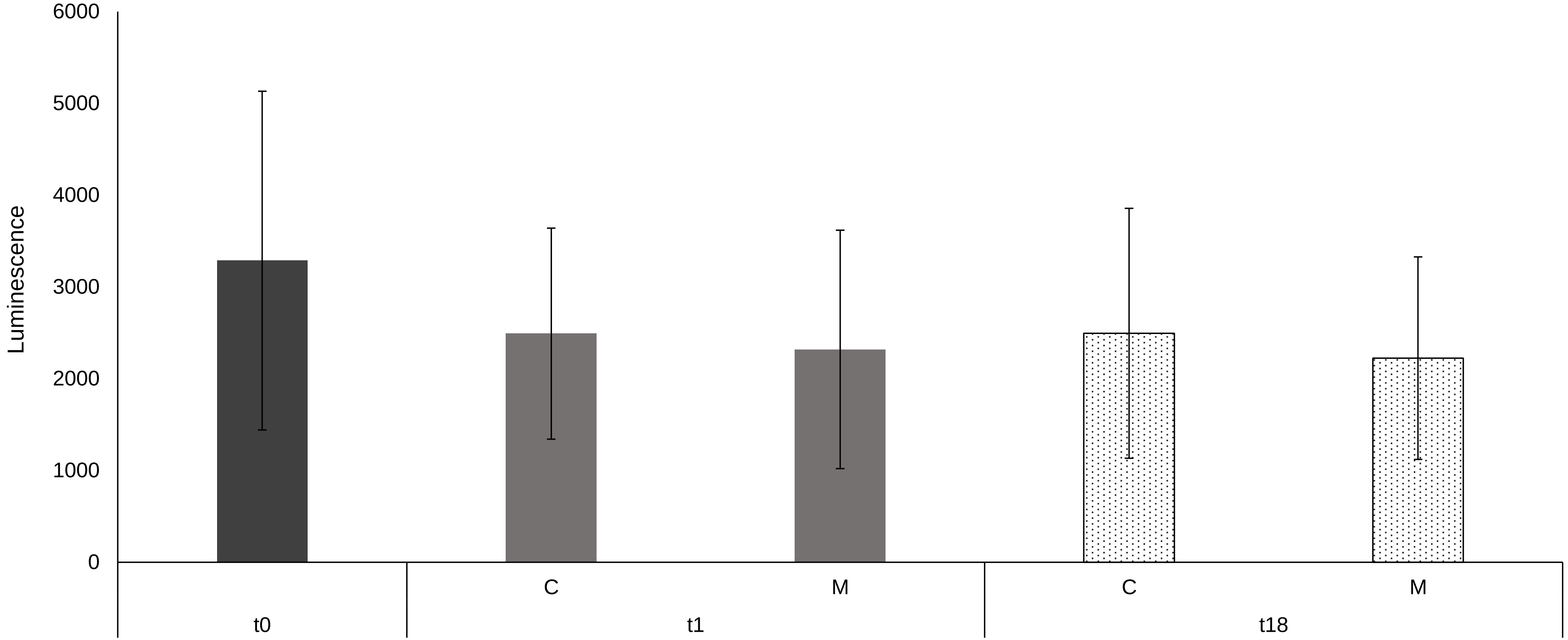

Quantification of the NF-κB pathway activation induced by the (un-) evolved *P. aeruginosa* strains after 4h of infection in the 3-D A549 lung epithelial model. Graphs show means of three independent lineages (L1, L2, L3), error bars indicate standard deviations. No significant differences. Neg. ctrl: negative control of uninfected cells. T0: timepoint 0, planktonic culture. T1: timepoint 1, 72h biofilm cells. T18: timepoint 18, biofilm cells after 18 cycles (54 days). C: control single-culture, M: microbiome.

Fig S8 O Cytotoxicity (annexin V-PI assay) - POOLED

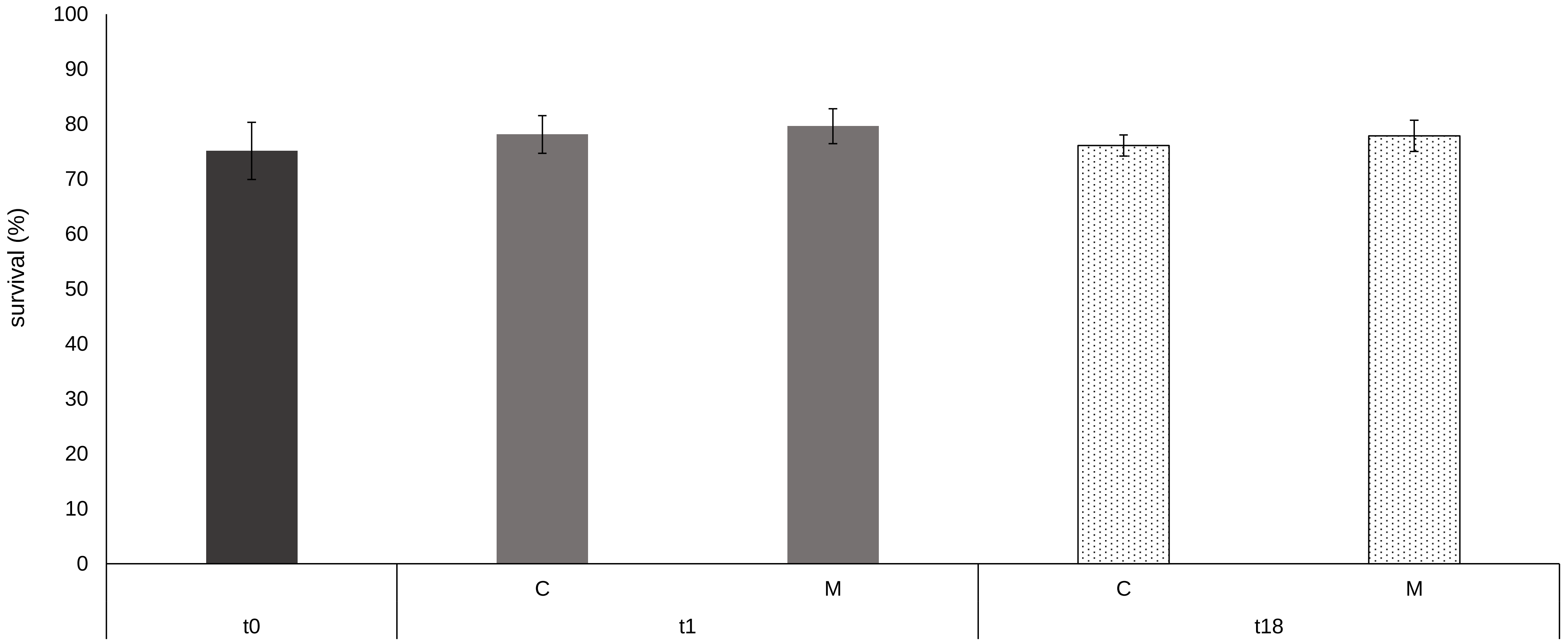

Cytotoxicity of (un-) evolved *P. aeruginosa* populations after 6h of infection in the 3-D A549 lung epithelial model. Graphs show means of three independent lineages (L1, L2, L3), error bars indicate standard deviations. No significant differences. Neg. ctrl: negative control of uninfected cells. T0: timepoint 0, planktonic culture. T1: timepoint 1, 72h biofilm cells. T18: timepoint 18, biofilm cells after 18 cycles (54 days). C: control single-culture, M: microbiome.

Fig S8 P

Competition experiment - POOLED

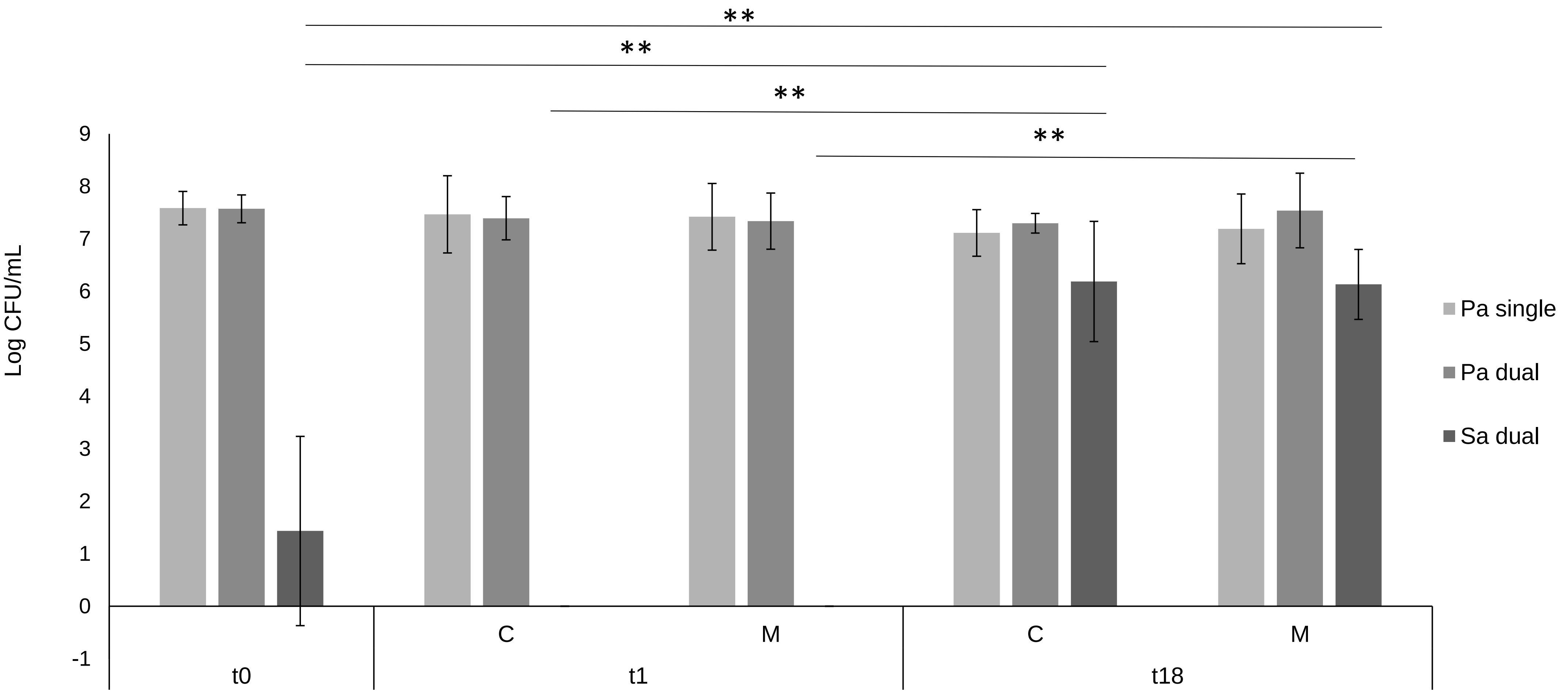

Determination of competition in dual-species biofilms of *P. aeruginosa* strains with *S. aureus* SP123. Pa single: *P. aeruginosa* strains as single-species biofilm, **Pa dual**: fraction of *P. aeruginosa* in the dual-species biofilm with *S. aureus*. **Sa dual**: fraction of *S. aureus* in the dual-species biofilm with *P. aeruginosa*. Graphs show means of three independent lineages (L1, L2, L3) and error bars indicate standard deviations. ** $p \leq 0.01$ (*S. aureus* T18 versus T0 and T1). T0: timepoint 0, planktonic start culture. T1: timepoint 1, 72h biofilm cells. T18: timepoint 18, biofilm cells after 18 cycles (54 days). C: control single-culture. M: microbiome.

Table S1. Overview of strains and culturing conditions used in this study.

Species name	Strain number	Isolation site	Liquid culture medium	Solid medium
Pseudomonas aeruginosa	AA2 = LMG27630	CF lung (early infection) [35]	BHI/LB	LB
Staphylococcus aureus	SP123	Sputum (Belgium) [36]	BHI	LB
Streptococcus anginosus	LMG14696	Respiratory tract	BHI	BHI
Achromobacter xylosoxidans	LMG26680	Sputum CF patient (Belgium)	BHI	NA
Rothia mucilaginosa	DSM20746	Throat	BHI	NA
Gemella haemolysans	LMG18984	Sputum	BHI	CBA

BHI: Brain Heart Infusion broth/agar; LB: Luria Bertani broth/agar; NA: Nutrient agar; CBA: Columbia Blood Agar (Columbia agar base + 5% sheep blood).

Table S2. Primer sequences used for Sanger sequencing in *P. aeruginosa*.

Target gene	Oligonucleotide sequence (5' – 3')
lasR	F: 5' – TGGGCTGACTGGACATCTTC – 3'<br>R: 5' – TTCGAGAATGGCGAGAACCT – 3'
pqsR	F: 5' – CGGGCGCTATCTATCGAGTC – 3'<br>R: 5' – TACCACTCGCTGGTGGTCAG – 3'

Table S3. Whole genome sequencing data at different time points during evolution (T1, T10, T18) of *P. aeruginosa* compared to the starting planktonic culture strain (T0). In: insertion (+), Del: deletion (Δ), SNP: single nucleotide polymorphism (→). nt: nucleotide. L1, L2, L3: lineage 1, 2, 3. C: control (= evolution in the absence of the microbiome), M: microbiome (= evolution in the presence of the microbiome). PA number: *Pseudomonas aeruginosa* PAO1 homologue number. This table lists genes that were mutated in at least one sample at a frequency above 35%.

Gene name	Nucleotides	Position (contig) nt	T0	T1 L1 C	T10 L1 C	T18 L1 C	T1 L1 M	T10 L1 M	T18 L1 M	T1 L2 C	T10 L2 C	T18 L2 C	T1 L2 M	T10 L2 M	T18 L2 M	T1 L3 C	T10 L3 C	T18 L3 C	T1 L3 M	T10 L3 M	T18 L3 M
upstream *sahH*	G → A	95660	-	-	77	99	-	-	56	-	-	-	-	-	-	-	11	77	-	-	10
upstream *sahH*	ΔG	95633	-	-	-	-	-	-	-	-	-	32	-	-	-	-	-	-	-	-	-
lasR	+A	294001	3	37	99	100	35	100	100	58	92	94	51	28	58	33	78	89	33	5	12
lasR	ΔG	294159	30	65	-	-	56	-	-	32	3	-	43	60	31	64	22	10	58	96	89
pilA	ΔCTACCA	118729	-	-	71	97	-	81	92	-	75	98	-	77	49	-	61	87	-	65	93
pqsR	+A	20879	-	-	87	80	-	2	11	-	-	-	-	-	-	-	-	-	-	-	-
pqsR	transposon	20654	-	-	6	5	-	-	-	-	-	-	-	-	-	-	-	-	-	-	-
pqsR	ΔTTGAT	20762	-	-	-	-	-	62	69	-	-	-	-	-	-	-	-	-	-	-	-
pqsR	12 nt deletion	21061	-	-	-	-	-	9	7	-	-	-	-	-	-	-	-	-	-	-	-
pqsR	C → T	20505	-	-	-	-	-	-	-	-	37	38	-	-	-	-	-	-	-	-	-
pqsR	A → T	21047	-	-	-	-	-	-	-	-	-	-	-	17	59	-	-	-	-	-	-
pqsA-phnA	large deletion	13454	-	-	-	-	-	-	-	-	-	-	-	-	10	-	-	-	-	-	-
pqsR	+G	20642	-	-	-	-	-	-	-	-	-	-	-	-	-	-	64	74	-	-	-
pqsR	T → G	20645	-	-	-	-	-	-	-	-	-	-	-	-	-	-	4	9	-	-	-
pqsR	transposon	21137	-	-	-	-	-	-	-	-	-	-	-	-	-	-	-	-	-	47	33
pqsR	ΔAG	20400	-	-	-	-	-	-	-	-	-	-	-	-	-	-	-	-	-	4	5
mexR	G → A	221690	-	-	-	22	-	-	-	-	-	-	-	-	-	-	-	-	-	-	-
mexR	35 nt duplication	222153	-	-	-	-	-	-	-	-	-	24	-	-	-	-	-	-	-	-	-
mexR	T → G	221786	-	-	-	-	-	-	-	-	-	17	-	-	-	-	-	-	-	-	-
mexR	C → A	222105	-	-	-	-	-	-	-	-	-	12	-	-	-	-	-	-	-	-	-
mexR	G → C	221695	-	-	-	-	-	-	-	-	-	-	-	-	23	-	-	-	-	-	-
mexR	T → G	222042	-	-	-	-	-	-	-	-	-	-	-	-	-	-	-	46	-	-	-
mexR	G → A	221744	-	-	-	-	-	-	-	-	-	-	-	-	-	-	3	9	-	-	-
mexR	G → T	221842	-	-	-	-	-	-	-	-	-	-	-	-	-	-	-	6	-	-	-
gntR	C → G	41825	-	-	-	41	-	-	-	-	-	-	-	-	-	-	-	-	-	-	-
gntR	A → C	41176	-	-	-	4	-	-	-	-	-	-	-	-	-	-	-	-	-	-	-
gntR	G → A	40992	-	-	-	2	-	-	-	-	-	-	-	-	-	-	-	-	-	-	-
gntR	A → C	41988	-	-	-	-	-	-	-	-	-	-	-	-	-	-	-	9	-	-	-
gntR	+CTG	41357	-	-	-	-	-	-	-	-	-	-	-	-	-	-	-	9	-	-	-
gntR	+C	40926	-	-	-	-	-	-	-	-	-	-	-	-	-	-	-	2	-	-	-

Table S4. Mutations occurring at a frequency of ≤35% in *P. aeruginosa* AA2 populations from T18.

Contig	Position		Frequency (%)	Lineage	PA number	Annotation
15	392887	SNV, T→C, V→V	7	T18 L1 Control	PA1874	Hypothetical protein
34	33592	SNV, C→A, L→L	31	T18 L1 Control	PA4125	5-carboxymethyl-2-hydroxymuconate delta-isomerase, hpcD
19	56465	SNV, A→C	8	T18 L1 Mix	PA3246	3'UTR of rluA, Ribosomal large subunit pseudouridine synthase A
11	105689..105704	16 nt Deletion, CGCATCGCCCAGCCGC	6	T18 L2 Control	PA1085	Flagellar protein, FlgJ
17	250042	SNV, C→T, R→L	18	T18 L2 Control	PA2824	Surface attachment and growth sensor hybrid, SagS
15	200522..200527	6 nt deletion, GTTGGC	9	T18 L2 Mix	PA1695	Type III secretion protein, pscP
15	200591..200596	6 nt deletion, GGCGTT	19	T18 L2 Mix	PA1695	Type III secretion protein, pscP
15	232331	SNV, G→A, P→L	6	T18 L2 Mix	PA1734	Cytochrome c, mono- and diheme binding
11	138598..138609	12 nt deletion, TGACGCAGCTTC	6	T18 L4 Mix	PA1103	Flagellar assembly protein, FliH
19	89564..89574	11 nt deletion GTCCTCGGCGC	9	T18 L4 Mix	PA3271	Sensory box histidine kinase/response regulator

Table S5. Sanger sequencing data for *lasR* and *pqsR* in ten isolates (single colonies: C1 – C10) from strain T18 L2 ctrl.

isolate	***lasR*** insertion	***pqsR*** SNP:G → A (reverse complement)
**C1**	ACTCGGCGCGCTGAAG	TCGTGGAAAATTTCGACAA
**C2**	ACTCGGCGCGCTGAAG	TCGTGGAAAATTTCGACAA
**C3**	ACTCGG**T**GC**T**CTGAAG	TCGTGGAAAATTTCGACGA (no SNV)
**C4**	ACTCGGCGCGCTGAAG	TCGTGGAAAATTTCGACGA (no SNV)
**C5**	ACTCGGCGCGCTGAAG	TCGTGGAAAATTTCGACGA (no SNV)
**C6**	ACTCGGCGCGCTGAAG	TCGTGGAAAATTTCGACAA
**C7**	ACTCGGCGCGCTGAAG	TCGTGGAAAATTTCGACAA
**C8**	ACTCGGCGCGCTGAAG	TCGTGGAAAATTTCGACAA
**C9**	ACTCGGCGCGCTGAAG	TCGTGGAAAATTTCGACGA (no SNV)
**C10**	ACTCGGCGCGCTGAAG	TCGTGGAAAATTTCGACAA

Table S6. MIC values of (un-)evolved *P. aeruginosa* strains in lineages 1, 2, and 3 (L1, L2, L3).

**L1**	**ceftazidime**		**ciprofloxacin**		**colistin**		**tobramycin**		**aztreonam**	
	ctrl	microb	ctrl	microb	ctrl	microb	ctrl	microb	ctrl	microb
**T0**	4		0.25		8		1		4	
**T1**	16	32	0.25	0.25	4	8	1	1	16	16
**T18**	16	16	0.25	0.25	4	8	1	1	>64	16
**L2**	ceftazidime		ciprofloxacin		colistin		tobramycin		aztreonam	
	ctrl	microb	ctrl	microb	ctrl	microb	ctrl	microb	ctrl	microb
**T0**	4		0.25		8		1		4	
**T1**	16	8	0.25	0.25	8	4	1	1	8	16
**T18**	32	16	0.5	0.5	8	8	1	1	8	16
**L3**	**ceftazidime**		**ciprofloxacin**		**colistin**		**tobramycin**		**aztreonam**	
	ctrl	microb	ctrl	microb	ctrl	microb	ctrl	microb	ctrl	microb
**T0**	4		0.25		8		1		4	
**T1**	8	8	0.25	0.25	8	4	1	1	8	8
**T18**	16	8	0.25	0.25	8	8	1	1	32	4
